# Factors influencing immediate post-angiographic occlusion outcomes in intracranial aneurysms treated with the woven endobridge device: a multi-center analysis and predictive model from the WorldWideWEB consortium

**DOI:** 10.1007/s10143-025-03928-w

**Published:** 2025-12-02

**Authors:** Muhammed Amir Essibayi, Mohamed Sobhi Jabal, Hasan Jamil, Hamza Adel Salim, Basel Musmar, Nimer Adeeb, Mahmoud Dibas, Nicole M. Cancelliere, Jose Danilo Bengzon Diestro, Oktay Algin, Sherief Ghozy, Sovann V. Lay, Adrien Guenego, Leonardo Renieri, Joseph Carnevale, Guillaume Saliou, Panagiotis Mastorakos, Kareem El Naamani, Arbaz A. Momin, Eimad Shotar, Markus Möhlenbruch, Michael Kral, Charlotte Chung, Mohamed M. Salem, Ivan Lylyk, Paul M. Foreman, Hamza Shaikh, Vedran Župančić, Muhammad U. Hafeez, Joshua Catapano, Muhammad Waqas, Muhammed Said Besler, Yasin Celal Gunes, James D. Rabinov, Julian Maingard, Clemens M. Schirmer, Mariangela Piano, Anna L. Kühn, Caterina Michelozzi, Robert M. Starke, Ameer Hassan, Mark Ogilvie, Anh Nguyen, Jesse Jones, Waleed Brinjikji, Marie T. Nawka, Marios Psychogios, Christian Ulfert, Bryan Pukenas, Jan-Karl Burkhardt, Thien Huynh, Juan Carlos Martinez-Gutierrez, Sunil A. Sheth, Diana Slawski, Rabih Tawk, Benjamin Pulli, Boris Lubicz, Pietro Panni, Ajit S. Puri, Guglielmo Pero, Eytan Raz, Christoph J. Griessenauer, Hamed Asadi, Adnan Siddiqui, Elad I. Levy, Deepak Khatri, Neil Haranhalli, Andrew F. Ducruet, Felipe C. Albuquerque, Robert W. Regenhardt, Christopher J. Stapleton, Peter Kan, Vladimir Kalousek, Pedro Lylyk, Srikanth Boddu, Jared Knopman, Stavropoula I. Tjoumakaris, Hugo H. Cuellar-Saenz, Pascal M. Jabbour, Frédéric Clarençon, Nicola Limbucci, Vitor Mendes Pereira, Aman B. Patel, David J. Altschul, Adam A. Dmytriw

**Affiliations:** 1 Departments of Neurological Surgery, Radiology, and Montefiore-Einstein Cerebrovascular Research Lab, Montefiore Medical Center, Albert Einstein College of Medicine, Bronx, NY USA; 2 Department of Radiology, Division of Neuroradiology, Johns Hopkins Medical Center, Baltimore, MD USA; 3 Department of Neurosurgery and Interventional Neuroradiology, Louisiana State University, Shreveport, LA USA; 4 Neurovascular Centre, Divisions of Therapeutic Neuroradiology and Neurosurgery, St. Michael’s Hospital, University of Toronto, Toronto, ON Canada; 5 Department of Radiology, Medical Faculty of Ankara University, Ankara, Turkey; 6 Departments of Radiology and Neurosurgery, Mayo Clinic, Rochester, MN USA; 7 Department of Neuroradiology, Centre Hospitalier de Toulouse, Toulouse, France; 8 Department of Neuroradiology, Hôpital Universitaire Erasme, Brussels, Belgium; 9 Department of Neuroradiology, Ospedale Careggi Di Firenze, Florence, Italy; 10 Department of Neurosurgery and Neuroradiology, New York Presbyterian Hospital and Weill Cornell School of Medicine, New York, NY USA; 11 Department of Neuroradiology, Centre Hospitalier Vaudois de Lausanne, Lausanne, Switzerland; 12 Department of Neurosurgery, Thomas Jefferson University Hospital, Philadelphia, PA USA; 13 Department of Neuroradiology, Hôpital Pitié-Salpêtrière, Paris, France; 14 Department of Neuroradiology, Universitätsklinikum Heidelberg, Heidelberg, Germany; 15 Department of Neurosurgery, Christian Doppler University Hospital & Institute of Neurointervention, Salzburg, Austria; 16 Departments of Radiology & Neurosurgery, NYU Langone Health Center, New York, NY USA; 17 Department of Neurosurgery, University of Pennsylvania Medical Center, Philadelphia, PA USA; 18 Department of Neuroradiology, Clínica La Sagrada Familia, Buenos Aires, Argentina; 19 Department of Neurosurgery, Orlando Health Neuroscience and Rehabilitation Institute, Orlando, FL USA; 20 Department of Neuroradiology, Clinical Hospital Center ‘Sisters of Mercy’, Zagreb, Croatia; 21 Department of Neurosurgery, UTMB and Baylor School of Medicine, Houston, TX USA; 22 Department of Neurosurgery, Barrow Neurological Institute, Phoenix, AZ USA; 23 Department of Neurosurgery, State University of New York at Buffalo, Buffalo, NY USA; 24 Department of Radiology, Kahramanmaraş Necip Fazıl City Hospital, Kahramanmaraş, Turkey; 25 Department of Radiology, Kırıkkale Yuksek Ihtisas Hospital, Kırıkkale, Turkey; 26 Neuroendovascular Program, Massachusetts General Hospital, Harvard University, Boston, MA USA; 27 Department of Neuroradiology, Austin Health, Victoria, Australia; 28 Department of Neurosurgery and Radiology, Geisinger Hospital, Danville, PA USA; 29 Department of Neuroradiology, Ospedale Niguarda Cà Granda, Milan, Italy; 30 Department of Neuroradiology, UMass Memorial Hospital, Worcester, MA USA; 31 Department of Neuroradiology, Ospedale San Raffaele, Milan, Italy; 32 Department of Neurosurgery, University of Miami, Miami, FL USA; 33 Department of Neuroradiology, Valley Baptist Neuroscience Institute, Harlingen, TX USA; 34 Deparments of Neurosurgery and Radiology, University of Alabama at Birmingham, Birmingham, AL USA; 35 Department of Diagnostic and Interventional Neuroradiology, University Medical Center Hamburg-Eppendorf, Hamburg, Germany; 36 Department of Neuroradiology, University Hospital of Basel, Basel, Switzerland; 37 Departments of Radiology and Neurosurgery, Mayo Clinic, Jacksonville, FL USA; 38 Department of Neuroradiology, University of Texas Health Science Center at Houston, Houston, USA; 39 Department of Radiology, Division of Neuroimaging and Neurointervention, Stanford University School of Medicine, Stanford, CA United States

**Keywords:** Aneurysms, Brain, Woven EndoBridge, Immediate occlusion

## Abstract

The Woven EndoBridge (WEB) device treats wide-necked bifurcation aneurysms, but occlusion rates vary. This study aims to identify factors associated with immediate WEB device occlusion. Data from patients treated with WEB devices across 36 sites were analyzed. Machine learning algorithms and ordinal regression models were developed to predict immediate incomplete occlusion for ruptured and unruptured aneurysms. The study included 1565 patients, with 436 ruptured and 1129 unruptured aneurysms. Immediate complete occlusion was achieved in 38.3% of ruptured and 32.8% of unruptured aneurysms. For ruptured aneurysms, the CatBoost classifier achieved an AUROC of 0.69. Key predictors of incomplete occlusion included pretreatment mRS, aneurysm diameter, and MCA location. Ordinal regression revealed that smoking history (OR: 1.95, *p* < 0.001), neck diameter (Odds Ratio [OR]: 1.50, *p* < 0.001), and presence of a branch from the aneurysm (OR: 2.06, *p* = 0.016) were associated with incomplete, while bifurcation aneurysms (OR: 0.55, *p* = 0.017) were associated with complete immediate occlusion. For unruptured aneurysms, the CatBoost classifier achieved an AUROC of 0.68. Significant predictors of immediate incomplete occlusion included aneurysm neck width, MCA location, and presence of daughter sac. Ordinal regression revealed that smoking history (OR: 1.29, *p* = 0.032), neck diameter (OR: 1.24, *p* < 0.001), and presence of a daughter sac (OR: 1.53, *p* = 0.005) were associated with incomplete, while bifurcation aneurysms (OR: 0.71, *p* = 0.02) and posterior circulation location (OR: 0.68, *p* = 0.01) were associated with complete immediate occlusion. Careful evaluation of patient demographics and specific aneurysm characteristics may help improve the outcomes of intracranial aneurysms treated with WEB device.

**Supplementary Information:**

The online version contains supplementary material available at 10.1007/s10143-025-03928-w.

## Introduction

The Woven EndoBridge (WEB) device is a novel intrasaccular aneurysm treatment option that has been developed as an alternative to traditional methods such as endovascular coiling and neurosurgical clipping for the treatment of wide-necked bifurcation aneurysms [1]. The WEB device disrupts the inflow of blood within the aneurysm sac by deploying an intra-aneurysmal mesh implant and subsequently promotes occlusion through endothelialization [2]. Since its introduction, the WEB device has increasingly been adopted to treat wide-necked, bifurcation, and morphologically complex intracranial aneurysms [2, 3]. The WEB-IT trial, a key study, demonstrates the long-term safety and efficacy of the WEB device for wide-neck bifurcation aneurysms [4, 5]. Despite its growing use, studies have reported varying rates of complete aneurysm occlusion following WEB implantation, suggesting that the determinants of successful outcomes still need to be fully understood [2, 6–9].

Achieving immediate post-angiographic complete aneurysm occlusion is particularly critical in the context of ruptured aneurysms, [10], where the risk of rebleeding is high and potentially fatal. There is a notable gap in the literature concerning the specific factors that influence the effectiveness of WEB devices in achieving immediate post-angiographic occlusion, particularly ruptured aneurysms. A recent meta-analysis by Pineda-Castillo reported a 27.8% rate of immediate complete occlusion following the deployment of the WEB device in unruptured aneurysms across six studies. However, this meta-analysis did not evaluate ruptured aneurysms or identify predictors for this outcome [11]. Thus, a deeper understanding of the demographic, clinical, morphological, and procedural variables that impact WEB occlusion outcomes is essential for guiding patient selection and optimizing treatment strategies in ruptured and unruptured aneurysms.

This multi-center international study aims to identify factors influencing immediate post-angiographic occlusion outcomes of ruptured and unruptured intracranial aneurysms.

## Materials and methods

### Patient dataset and variables

A retrospective compilation of data was undertaken by the WorldWideWeb consortium, encompassing contributions from 36 hospitals across North and South America, Asia, Europe, and Australia. The consortium conducted continuous updates of patient outcomes and the registration of new cases. Approval from the Institutional Review Board (IRB) was secured at each participating institution, and patient consent was waived due to the retrospective nature design. Included were adult individuals aged 18 years and older who had been treated with WEB devices for intracranial saccular aneurysms from January 2011 to December 2022. The scope of the study was not confined by the aneurysm's location or its state of rupture.

The assortment of variables compiled encompassed demographic information (age, gender, smoking status), specifics of aneurysm features (including location, neck width, maximal diameter, height, width, existence of a daughter sac, emergence of a branch from the dome, bifurcation site, any antecedent treatment, and the degree of thrombosis), presentation attributes (symptoms and a history of subarachnoid hemorrhage [SAH]), and the urgency of treatment, which was stratified into four temporal categories: elective, chronic (less than two weeks), subacute (greater than two weeks to 24 h), and acute (within 24 h). Smoking status was evaluated as an ordinal and categorical (2 and 3 categories) variable. The Smoking Intensity Scale was defined as an ordinal variable categorizing smoking status into never smokers, former smokers, and current smokers, thus reflecting increasing levels of smoking exposure. Additionally, a binary categorization of smoking history (never vs. ever smokers) was used for specific analyses. Maximal aneurysm diameter was defined as the largest dimension of the aneurysm, whether it was width, height, or depth. Angiographic assessments were recorded, detailing immediate and follow-up Raymond-Roy Occlusion Classification (RROC) grades and occlusion statuses, including retreatment procedures. The procedural specifics were documented, covering the access route, adjunct device utilization, and complications, alongside clinical results such as the Hunt-Hess and pre-treatment modified Rankin Scale (mRS) grades. The application of the WEB device was designated for cases treating aneurysms situated in both on and off-label locations.

This analysis's principal outcome of interest was the attainment of immediate post-angiographic occlusion outcome. Immediate post-angiographic complete occlusion was defined as an RROC grade of 1. Instances of residual neck filling and residual aneurysm (RROC = 2,3) were denoted as incomplete occlusion. Adequate occlusion was defined as RROC = 1,2. Other variables cataloged included the timing of the subarachnoid hemorrhage event, smoking history, categorization of aneurysm size, and the outcomes of occlusion.

### Statistical analysis

Differences in demographic and clinical characteristics between the two patient groups were analyzed using SciPy version 1.6.2, integrated with Python. All variables were analyzed based on the immediate post-angiographic occlusion outcome and aneurysm rupture status. Continuous variables were described using medians and interquartile ranges (IQR), while categorical variables were represented as proportions. Univariate analyses were conducted to compare the groups concerning adverse outcomes. The Kruskal–Wallis test was applied to continuous, non-normally distributed variables, and the Chi-Square test was used for categorical variables. For the purposes of modeling, missing data were imputed to maintain analytical integrity. Continuous variables were imputed utilizing the mean value, whereas categorical variables with less than 20% missingness were imputed using the mode. Variables exhibiting greater than 20% missingness were excluded from the regression and machine learning models.

#### Machine learning modeling and interpretation

Python 3.9 was utilized for processing and modeling the collected features. The features were normalized using min–max scaling to ensure their values ranged from 0 to 1, optimizing them for the learning algorithms. Dimensionality reduction was achieved through the application of Maximum Relevance—Minimum Redundancy (MRMR), which selected the top 25% of the most pertinent features to streamline the model's interpretability.

Machine learning algorithms were deployed to forecast the likelihood of unfavorable outcomes based on the refined features. The data was divided into a training set (75%) and a test set (25%), with model validation performed through tenfold cross-validation within the training set. The models developed included Decision Tree, Gaussian Naïve Bayes, Multilayer Perceptron, K-Nearest Neighbors, Random Forest, Bagging Classifier, Gradient Boosting, and CatBoost classifier. Hyperparameters were optimized using a grid-search strategy. The models' performance was evaluated on the test set across several metrics: area under the receiver operating characteristic curve (AUROC), accuracy, F1 score, precision, and recall. The most effective model was further analyzed using Shapley Additive explanations (SHAP). to understand the impact of features on the predictions. Interpretations of the predictions on the test set, alongside individual assessments using force plots, provided clinical insights into the factors contributing to the prediction of occlusion status following WEB device deployment.

#### Ordinal regression

To identify factors associated with immediate post-angiographic RROC, we developed ordinal regression models incorporating variables such as pre-treatment mRS score, age, gender, bifurcation aneurysm status, neck diameter, maximal diameter, branch presence, history of smoking, circulation type, prior treatment, aneurysm height, aneurysm width, daughter sac presence, adjunct device use, and WEB device type. Adjusted odds ratio (aOR) and 95% confidence intervals (CI) were reported. We fitted a multilevel ordinal regression using Cumulative Link Mixed Models (CLMM) with a random intercept for institutions using the Laplace approximation to account for institutional variability. All analyses were conducted in R version 4.3.3, using the MASS and Ordinal packages.

## Results

### Patient cohort

**Fig. 1:**
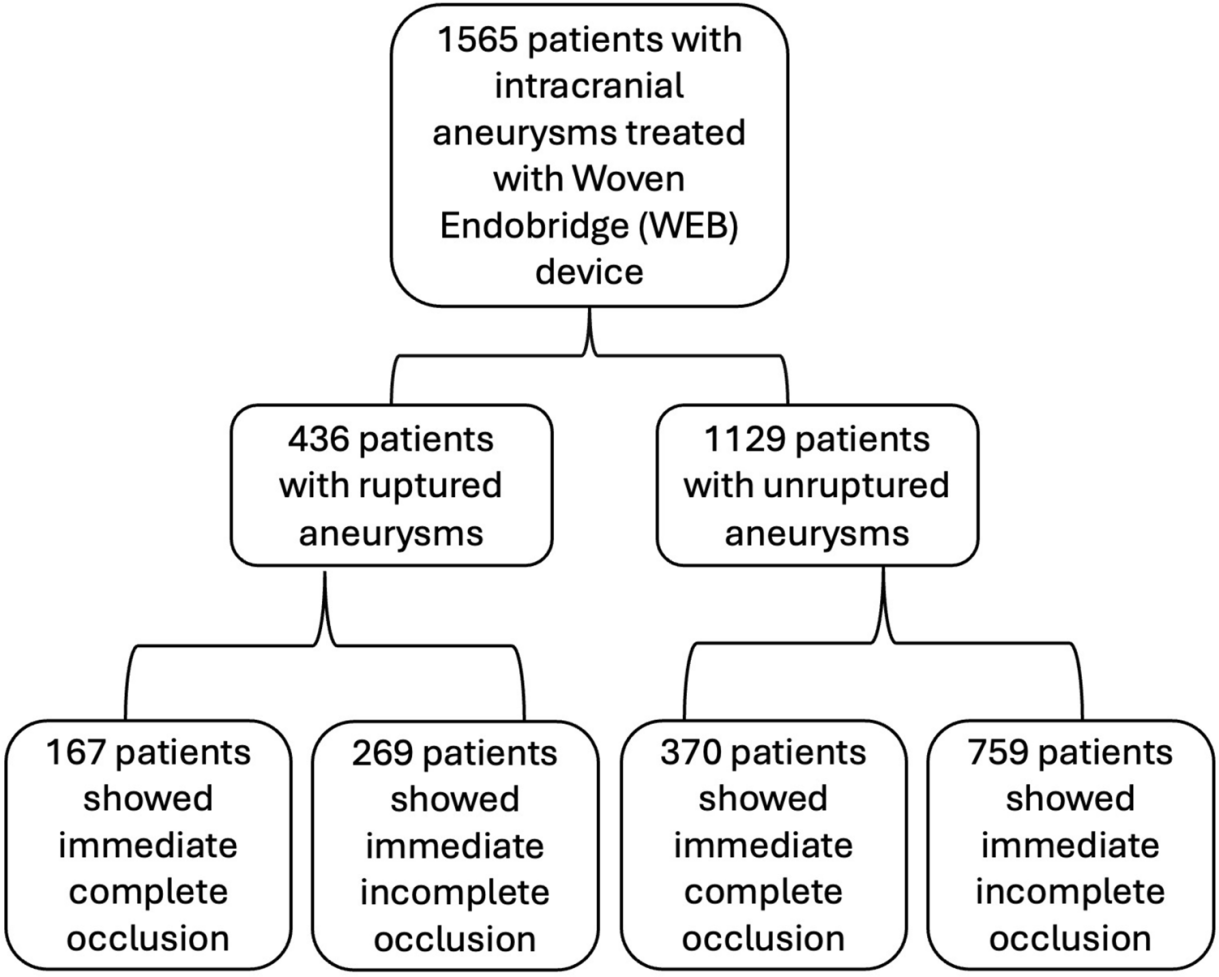
Flowchart shows the study cohort and its outcome subgroups

A total of 1565 patients (436 patients with ruptured and 1129 patients with unruptured aneurysms) underwent WEB device treatment for aneurysm occlusion (Fig. 1). Among these, 1028 (66%) had an immediate post-angiographic incomplete occlusion, while 537 (34%) achieved immediate post-angiographic complete occlusion. Immediate post-angiographic adequate occlusion was observed in 846 (54%) of patients.

When comparing immediate post-angiographic complete and incomplete occlusion groups (Table 1), the incomplete occlusion group was slightly older (median age 60 vs. 59 years, *p* = 0.038). The smoking intensity scale differed significantly between the two groups (*p* = 0.001). Current smokers comprised 31.1% overall (31.7% in the incomplete occlusion group vs. 29.8% in the complete occlusion group, *p* < 0.001), former smokers 21.2% overall (24.5% vs. 14.7%, *p* < 0.001), and never smokers 47.8% overall (43.8% vs. 55.5%, *p* < 0.001). The complete occlusion group had higher rates of hemorrhagic presentation (*p* = 0.03) and multiple aneurysms (*p* < 0.001). Aneurysms in the incomplete occlusion group were characterized by significantly larger neck diameter (median 4 mm vs. 3.6 mm, *p* < 0.001), greater maximal diameter (median 6.6 vs. 6 mm, *p* < 0.001), and increased width (median 5.5 vs. 5 mm, *p* < 0.001). This group also showed a higher prevalence of daughter sacs (*p* < 0.001) and aneurysms with branches arising from the sac (*p* < 0.001), while there was a lower prevalence of bifurcation aneurysms (*p* = 0.002). At the last follow-up, the incomplete occlusion group had worse median mRS scores (2 vs. 1, *p* < 0.001) (Table 2). Gender distribution and length of last follow-up were similar between groups.

**Table 1:** Baseline characteristics of patients

Variable name	All 1565	Immediate post-angiographic incomplete occlusion 1028 (66%)	Immediate post-angiographic complete occlusion 537 (34%)	*P* value
	*n* (%)	*n* (%)	*n* (%)	
Age (years), median (IQR)	60.0 (52.0, 68.0)	60.0 (52.0, 68.0)	59.0 (51.0, 67.0)	0.04
Gender (Male)	70.6% (1105)	70.4% (724)	70.9% (381)	0.87
Smoking				
Smoking Intensity Scale [Ordinal^#^], median (IQR)	1 (0.0, 2.0)	1 (0.0, 2.0)	0 (0.0,2.0)	0.001
Current	486 (31.1%)	326 (31.7%)	160 (29.8%)	< 0.001
Former	331 (21.2%)	252 (24.5%)	79 (14.7%)	
Never	748 (47.8%)	450 (43.8%)	298 (55.5%)	
History of Smoking	817 (52.2%)	578 (56.2%)	239 (44.5%)	< 0.001
Ruptured Aneurysms	436 (27.9%)	269 (26.2%)	167 (31.1%)	0.045
Pretreatment mRS, median (IQR)	0 (0.0, 2.0)	0.0 (0.0, 1.0)	0 (0.0,3.0)	0.03
Treatment Urgency Level^*^	0 (0.0, 2.0)	0.0 (0.0, 1.0)	0 (0.0,3.0)	0.03
Elective	1129 (72.1%)	759 (73.8%)	370 (68.9%)	0.15
> 2 weeks	24 (1.5%)	15 (1.5%)	9 (1.7%)	
< 2 weeks	36 (2.3%)	25 (2.4%)	11 (2%)	
Acute	376 (24%)	229 (22.3%)	147 (27.4%)	

^**#**^Smoking was treated as an ordinal variable in the order 0 “never smoker” | 1 “former smoker” | 2 “current smoker”

^*^Treatment urgency level was categorized in relation to treatment time in 4 groups as follows; elective, chronic (< 2 weeks), subacute (≥ 2 weeks – 24 h), and acute (within 24 h). This variable was treated as a continuous variable respectively in the order 0 | 1 | 2 | 3

Abbreviations: *IQR* Interquartile Range, *mRS* modified Rankin Scale

**Table 2:** Baseline characteristics of aneurysms treated with WEB device and technical characteristics of treatment

Variable name	All 1565	Immediate post-angiographic incomplete occlusion 1028 (66%)	Immediate post-angiographic complete occlusion 537 (34%)	*P* value
	*n* (%)	*n* (%)	*n* (%)	
Location				
Acom	494 (31.6%)	299 (29.1%)	195 (36.3%)	** < 0.001**
AICA	1 (0.1%)	1 (0.1%)	0 (0.0%)	>.99
Anterior choroidal	8 (0.5%)	3 (0.3%)	5 (0.9%)	0.19
ICA	130 (8.3%)	72 (7.0%)	58 (10.8%)	**0.01**
MCA	443 (28.3%)	339 (33.0%)	104 (19.4%)	** < 0.001**
Ophthalmic	22 (1.4%)	14 (1.4%)	8 (1.5%)	>.99
PICA	27 (1.7%)	17 (1.7%)	10 (1.9%)	0.92
Pcom	100 (6.4%)	61 (5.9%)	39 (7.3%)	0.36
SCA	13 (0.8%)	7 (0.7%)	6 (1.1%)	0.54
Basilar	249 (15.9%)	154 (15%)	95 (17.7%)	0.48
VA	6 (0.4%)	6 (0.6%)	0 (0.0%)	0.18
Anterior circulation	1269 (81.1%)	843 (82%)	426 (79.3%)	0.20
Posterior circulation	296 (18.9%)	185 (18%)	111 (20.7%)	
Aneurysm characteristics				
Aneurysm count (including nontreated)	0 (0.0,1.0)	0.0 (0.0,1.0)	0 (0.0,1.0)	< 0.001
Bifurcation aneurysm	1277 (81.6%)	816 (79.4%)	461 (85.8%)	0.002
Aneurysm maximal diameter (mm), median (IQR)	6.3 (5.0,8.0)	6.6 (5.3,8.1225)	6.0 (4.7,7.38)	< 0.001
< 10 mm	1357 (86.7%)	869 (84.5%)	488 (90.9%)	< 0.001
> 10 mm	208 (13.3%)	159 (15.5%)	49 (9.1%)	
Aneurysm height (mm), median (IQR)	5.7 (4.5,7.2)	5.715 (4.5,7.5)	5.5 (4.2,7.0)	0.01
Aneurysm width (mm), median (IQR)	5.3 (4.2,6.9)	5.5 (4.4,7.0)	5.0 (3.92,6.4)	< 0.001
Aneurysm neck (mm), median (IQR)	3.9 (3.0,4.8)	4.0 (3.1,5.0)	3.6 (3.0,4.1)	< 0.001
Daughter sac	377 (24.1%)	273 (26.6%)	104 (19.4%)	0.002
Branch from aneurysm	181 (11.6%)	143 (13.9%)	38 (7.1%)	< 0.001
Partially thrombosed aneurysm	23 (1.5%)	16 (1.6%)	7 (1.3%)	0.86
Technical characteristics				
Prior treatment	99 (6.3%)	72 (7.0%)	27 (5.0%)	0.16
WEB type				0.05
SL	1334 (85.2%)	885 (86.1%)	449 (83.6%)	
SLS	193 (12.3%)	114 (11.1%)	79 (14.7%)	
DL	38 (2.4%)	29 (2.8%)	9 (1.7%)	
Use of adjunct device	103 (6.6%)	59 (5.7%)	44 (8.2%)	0.08
Thromboembolic complications	105 (6.7%)	67 (6.5%)	38 (7.1%)	0.75

Abbreviations: *AICA* Anterior Inferior Cerebellar Artery, *DL* Dual-Layer, *ICA* Internal Carotid Artery, *IQR* Interquartile Range, *Pcom* Posterior Communicating Artery, *PICA* Posterior Inferior Cerebellar Artery, *SCA* Superior Cerebellar Artery, *SL* Single-Layer, *SLS* Single-Layer Spherical, *VA* Vertebral Artery

Significant differences in baseline characteristics were observed between the ruptured and unruptured aneurysms (Table 3). Patients with ruptured aneurysms had a higher mean pre-treatment mRS score (1.25 vs. 0.26, *p* < 0.001) and were younger (Median 57 years vs. 61 years, *p* < 0.001). The neck diameter was slightly smaller in the ruptured group (3.64 mm vs. 3.9 mm, *p* < 0.001), while the aneurysm width was also narrower (5.00 mm vs. 5.49 mm, *p* = 0.01). More males were in the ruptured group (37.61% vs. 26.22%, *p* < 0.001). Additionally, a higher percentage of ruptured aneurysms were located in the anterior circulation (85.55% vs. 79.36%, *p* = 0.01), and ruptured aneurysms were more likely to have a daughter sac (34.40% vs. 20.11%, *p* < 0.001).

**Table 3:** Baseline characteristics stratified by the rupture status

Variable	Ruptured aneurysms (*n* = 436)	Unruptured Aneurysms (*n* = 1129)	*P*-value
	*n* (%)	*n* (%)	
Demographics			
Pre-Treatment mRS Score, median (IQR)	0.00 [0.00, 2.00]	0.00 [0.00, 0.00]	< 0.001
Age (years), median (IQR)	57.00 [49.00, 64.00]	61.00 [53.00, 69.00]	< 0.001
Gender (Female)	272 (62.39%)	833 (73.78%)	< 0.001
History of Smoking	223 (51.15%)	594 (52.61%)	0.64
Aneurysm characteristics			
Bifurcation Aneurysm	356 (81.65%)	921 (81.58%)	> 0.99
Neck Diameter (mm), median (IQR)	3.64 [3.00, 4.40]	3.90 [3.10, 4.98]	< 0.001
Maximal Diameter (mm), median (IQR)	6.20 [5.00, 8.00]	6.40 [5.10, 8.00]	0.88
Branch from Aneurysm	48 (11.01%)	133 (11.78%)	0.73
Posterior Circulation	63 (14.45%)	233 (20.64%)	0.01
Aneurysm Height (mm), median (IQR)	5.70 [4.30, 7.05]	5.70 [4.50, 7.30]	0.73
Aneurysm Width (mm), median (IQR)	5.00 [4.00, 6.52]	5.49 [4.40, 6.90]	0.01
Daughter Sac	150 (34.40%)	227 (20.11%)	< 0.001
Treatment characteristics			
Prior Treatment	24 (5.50%)	75 (6.64%)	0.48
Use of Adjunct Device	27 (6.19%)	76 (6.73%)	0.79
WEB Device Type			0.01
SL	367 (84.17%)	967 (85.65%)	
SLS	65 (14.91%)	128 (11.34%)	
DL	4 (0.92%)	34 (3.01%)	
Immediate post-angiographic Raymond-Roy Classification			0.01
Complete occlusion	167 (38.30%)	370 (32.77%)	
Remnant neck	96 (22.02%)	213 (18.87%)	
Remnant aneurysm	173 (39.68%)	546 (48.36%)	

Abbreviations: *DL* Dual-Layer, *IQR* Interquartile Range, *mRS* modified Rankin Scale, *SL* Single-Layer, *SLS* Single-Layer Spherical

### Machine learning modeling and ordinal regression results

#### Immediate occlusion status among ruptured aneurysms

Of 436 ruptured aneurysms, 269 (61.7%) showed immediate incomplete occlusion, and 167 (38.3%) showed complete occlusion. Immediate adequate occlusion was achieved in 60% of ruptured aneurysms. Machine learning algorithms were developed using ten key minimally interdependent features to predict occlusion status. The CatBoost classifier performed best, achieving an AUROC of 0.69. Model performance comparisons are shown in Supplemental Fig. 1A, with a detailed performance matrix in Supplemental Fig. 1B. Supplemental Fig. 2 presents a SHAP summary plot and heatmap illustrating individual feature effects on predictive outcomes. Feature importance rankings are detailed in Supplemental Fig. 3. The most significant positive predictors for incomplete occlusion in descending order were pretreatment mRS, aneurysm width, MCA location, smoking status, aneurysm neck diameter, emanating branches, maximal aneurysm diameter, and multiple aneurysms. In contrast, negative predictors for incomplete occlusion were anterior communicating artery (Acom) location and bifurcation aneurysm.

On multivariable ordinal regression analysis (Table 4), higher pre-treatment mRS score (OR: 1.17, 95% CI: 1.06–1.31, *p* = 0.003), history of smoking (OR: 1.95, 95% CI: 1.33–2.88, *p* < 0.001), neck diameter (OR: 1.50, 95% CI: 1.24–1.82, *p* < 0.001), maximal diameter (OR: 1.26, 95% CI: 1.04–1.54, *p* = 0.021), and branch from aneurysm (OR: 2.06, 95% CI: 1.15–3.76, *p* = 0.016) positively predicted incomplete occlusion, while bifurcation aneurysms (OR: 0.55, 95% CI: 0.33–0.89, *p* = 0.017), aneurysm height (OR: 0.82, 95% CI: 0.71–0.95, *p* = 0.007), and use of an adjunct device (OR: 0.44, 95% CI: 0.19–0.98, *p* = 0.047) were negative predictors for incomplete occlusion among ruptured aneurysms treated with WEB. Multilevel analysis (Supplemental Table 1) confirmed smoking history (OR: 1.95, 95% CI: 1.19–3.19, *p* = 0.008) and neck diameter (OR: 1.35, 95% CI: 1.08–1.70, *p* = 0.008) as significant predictors of incomplete occlusion.

**Table 4:** Ordinal regression to predict worse immediate post-angiographic occlusion outcomes after WEB deployment stratified by rupture status

	Ruptured aneurysms (*n* = 436)		Unruptured aneurysms (*n* = 1129)	
	Odds ratio (95% CI)	*p*-value	Odds ratio (95% CI)	*p*-value
Demographics				
Pre-Treatment mRS Score	1.17 [1.06, 1.31]	**0.003**	0.89 [0.76, 1.05]	0.16
Age	1.01 [0.99, 1.02]	0.48	1.01 [0.99, 1.01]	0.22
Gender (Male)	0.88 [0.59, 1.30]	0.52	0.86 [0.66, 1.11]	0.23
History of Smoking	1.95 [1.33, 2.88]	** < 0.001**	1.29 [1.02, 1.62]	**0.03**
Aneurysm characteristics				
Bifurcation Aneurysm	0.55 [0.33, 0.89]	**0.017**	0.71 [0.52, 0.95]	**0.02**
Neck Diameter	1.50 [1.24, 1.82]	** < 0.001**	1.24 [1.12, 1.38]	** < 0.001**
Maximal Diameter	1.26 [1.04, 1.54]	**0.021**	1.11 [0.99, 1.25]	0.08
Branch from Aneurysm	2.06 [1.15, 3.76]	**0.016**	1.29 [0.90, 1.86]	0.17
Posterior Circulation	1.53 [0.87, 2.70]	0.14	0.68 [0.51, 0.91]	**0.01**
Aneurysm Height	0.82 [0.71, 0.95]	**0.007**	0.89 [0.81, 0.97]	**0.007**
Aneurysm Width	0.91 [0.76, 1.09]	0.30	0.99 [0.89, 1.10]	0.83
Daughter Sac	0.94 [0.63, 1.41]	0.76	1.53 [1.14, 2.06]	**0.005**
Treatment characteristics				
Prior Treatment	1.29 [0.59, 2.90]	0.53	1.13 [0.72, 1.79]	0.60
Use of Adjunct Device	0.44 [0.19, 0.98]	**0.047**	0.58 [0.37, 0.92]	**0.019**
WEB device type				
WEB Device Type (DL)	[Reference]		[Reference]	
WEB Device Type (SL)	1.59 [0.25, 10.2]	0.61	0.84 [0.41, 1.67]	0.63
WEB Device Type (SLS)	1.14 [0.17, 7.82]	0.89	0.68 [0.31, 1.44]	0.31

The dependent variable “Immediate post-angiographic occlusion status” has the following order 1 “complete occlusion” | 2 “neck remnant” | 3 “aneurysm remnant”

Abbreviations: *CI* Confidence Interval, *DL* Dual-Layer, *mRS* modified Rankin Scale, *SL* Single-Layer, *SLS* Single-Layer Spherical

#### Immediate occlusion status among unruptured aneurysms

**Fig. 2:**
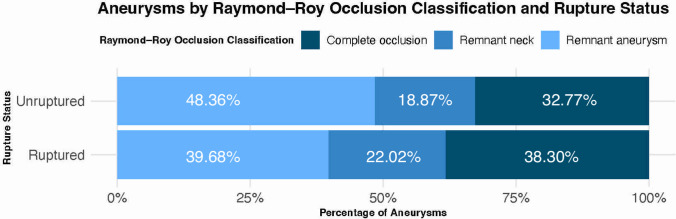
Distribution of Raymond-Ray occlusion classification grades of aneurysms treated with WEB device by their rupture status

Of 1129 unruptured aneurysms, 759 (67.2%) showed immediate post-angiographic incomplete occlusion, and 370 (32.8%) showed immediate complete aneurysm occlusion. The distribution of RROC grades by rupture status of aneurysms is illustrated in Fig. 2. Immediate post-angiographic adequate occlusion was achieved in 52% of unruptured aneurysms. Machine learning algorithms were developed using ten key minimally interdependent features to predict occlusion status. The CatBoost classifier performed best, achieving an AUROC of 0.68. Model performance comparisons are shown in Supplemental Fig. 4A, with a detailed performance matrix in Supplemental Fig. 4B. Supplemental Fig. 5 presents a SHAP summary plot and heatmap illustrating individual feature effects on predictive outcomes. Feature importance rankings are detailed in Supplemental Fig. 6. The most significant positive predictors for incomplete occlusion in descending order were aneurysm neck diameter, MCA location, presence of daughter sac, multiple aneurysms, maximal aneurysm diameter, and emanating branches. Negative predictors for incomplete occlusion were ICA and superior cerebellar artery locations.

On multivariable ordinal regression analysis **(**Table 4**)**, history of smoking (OR: 1.29, 95% CI: 1.02–1.62, *p* = 0.032), neck diameter (OR: 1.24, 95% CI: 1.12–1.38, *p* < 0.001), and presence of a daughter sac (OR: 1.53, 95% CI: 1.14–2.06, *p* = 0.005) positively predicted incomplete occlusion. However, bifurcation aneurysms (OR: 0.71, 95% CI: 0.52–0.95, *p* = 0.02), posterior circulation location (OR: 0.68, 95% CI: 0.51–0.91, *p* = 0.01), aneurysm height (OR: 0.89, 95% CI: 0.81–0.97, *p* = 0.007), and use of an adjunct device (OR: 0.58, 95% CI: 0.37–0.92, *p* = 0.019) were negative predictors for incomplete occlusion among unruptured aneurysms treated with WEB. Multilevel analysis (Supplemental Table 1) showed no significant associations with smoking history (OR: 0.88, 95% CI: 0.64–1.22, *p* = 0.45) and neck diameter (OR: 1.05, 95% CI: 0.93–1.18, *p* = 0.14) losing significance.

## Discussion

In this large-scale, multicenter, retrospective study involving 1565 patients, we explored the rates and predictors of immediate post-angiographic complete occlusion for both ruptured and unruptured aneurysms treated with WEB devices. We observed an overall rate of immediate post-angiographic complete occlusion of 34%, with 38.3% of ruptured aneurysms and 32.8% of unruptured aneurysms achieving complete occlusion. When including both Raymond-Roy grades I and II (adequate occlusion), the overall immediate adequate occlusion rate was 54%, comprising 60.32% in ruptured and 51.64% in unruptured aneurysms. Aneurysm morphological characteristics and a few patient-related characteristics, such as smoking history, were the most predictive of immediate post-angiographic incomplete occlusion.

These results are noteworthy when compared to previous studies evaluating traditional endovascular techniques. The ATENA study assessed unruptured aneurysms treated with coiling and remodeling techniques and reported a higher complete occlusion rate of 63% [12]. Similarly, the CLARITY study, which focused on ruptured aneurysms treated primarily with coiling, found a complete occlusion rate of 47.4% [10]. Immediate adequate occlusion rates (Raymond-Roy I–II) with coiling and stent-assisted coiling typically range from 80 to 95%, which are higher than those achieved with WEB at the time of deployment [13–18]. Although the WEB device may not improve immediate occlusion rates compared with traditional techniques, current evidence indicates its superior long-term durability due to its ability to promote delayed endothelization at the aneurysm neck, thereby enhancing the durability of treatment [6, 9, 19]. This distinction highlights that the WEB’s benefit lies primarily in sustained aneurysm stability rather than acute angiographic appearance.

Aneurysm morphology, specifically neck diameter, the presence of a daughter sac, and emanating branches, significantly predicted incomplete immediate occlusion. These findings are consistent with the broader literature [10, 12, 20], which has long recognized that aneurysms with wide necks, larger sizes, or complex configurations present more significant challenges to effective occlusion. The ATENA study similarly identified aneurysm size and neck configuration as critical determinants of occlusion quality, further validating the importance of these morphological factors [12]. On the other hand, posterior circulation aneurysms in our unruptured cohort were independently associated with a lower rate of immediate incomplete occlusions, consistent with previous findings that suggest the WEB device achieves stable occlusion in posterior circulation aneurysms, especially at bifurcation sites [21]. This stability may be due to the unique flow dynamics and morphology of posterior circulation aneurysms, which facilitate effective intrasaccular flow disruption by the WEB device. Additionally, the anatomic characteristics of posterior circulation aneurysms, such as smaller neck sizes and more favorable geometries for WEB deployment, likely contribute to these improved outcomes [22]. Although MCA aneurysms typically represent bifurcation aneurysms, they may exhibit distinct angioarchitectural features such as larger necks, wider domes, and higher branch incorporation rates, which make complete occlusion more challenging [23]. This may explain the contrasting findings where MCA location predicted incomplete occlusion, while bifurcation configuration overall favored better outcomes.

Interestingly, while some factors may vary in their influence on long-term occlusion outcomes with the WEB device [24, 25], specific aneurysmal morphological characteristics, such as aneurysm height and bifurcation location, resulted in better immediate occlusion outcomes. Bifurcation aneurysms are particularly well-suited for WEB deployment and optimal sizing [24]. Although aneurysm height is a well-known risk factor for aneurysm recurrence [25], its association with improved immediate occlusion outcomes using the WEB device is not fully understood and may benefit from further exploration. Initially, height was not identified as a significant predictor; however, adjusting for other cofactors became necessary, suggesting that height may play a role under specific conditions. A possible explanation is that greater aneurysm height might facilitate WEB oversizing, potentially making it a predictor of improved occlusion success in these particular aneurysm types.

In addition to morphological considerations, patient demographics such as age and smoking history were also found to influence occlusion outcomes, particularly in ruptured aneurysms. The CLARITY study showed that older age is linked to worse aneurysm occlusion outcomes, which was also evident in our cohort [10]. Specifically, older patients exhibited poorer occlusion outcomes, likely attributable to factors such as diminished vascular healing capacity, greater aneurysm complexity, an increased likelihood of recanalization, and a higher incidence of adverse events. These findings are consistent with the broader literature, which identifies advanced age as a significant predictor of incomplete aneurysm occlusion and subsequent treatment complications [26–29].

The distinct physiological and hemodynamic conditions in ruptured versus unruptured aneurysms significantly impact occlusion outcomes. In ruptured aneurysms, acute inflammation and altered hemodynamics may enhance the pro-thrombotic effects of the WEB device, leading to better clot formation. In contrast, the absence of these changes in unruptured aneurysms may reduce its efficacy [30, 31]. This was reflected in our study, where ruptured aneurysms had higher immediate occlusion rates. Regarding patient characteristics, smokers showed unfavorable outcomes, possibly due to smoking’s inhibitory effect on tissue A1AT, which usually regulates elastase activity. This disruption leads to increased elastin breakdown and reduced vessel elasticity, potentially diminishing the immediate effectiveness of the WEB device [32]. Despite using endothelialization devices like the WEB, these adverse effects persist and are independent of rupture status [33]. Smoking represents a critical risk factor that negatively impacts immediate occlusion outcomes, particularly in ruptured aneurysms, with the severity of risk increasing with closer proximity to smoking exposure. These findings are consistent with prior studies [34]. For instance, Mastorakos et al. identified multiple predictors of WEB occlusion in a multicenter setting, including a history of smoking and the presence of an aneurysm wall branch [34].

Our study utilized machine learning to improve the prediction of WEB device outcomes, capturing complex interactions between aneurysm shape and patient factors. The CatBoost classifier had an AUROC of about 0.69, showing moderate accuracy in a diverse, multi-center dataset where variability limits performance. It identified key predictors like pretreatment mRS score, aneurysm width, and bifurcation status. ML revealed nuanced, multi-factorial predictors beyond traditional logistic regression, offering a comprehensive view of factors influencing immediate occlusion. These models could help clinicians preoperatively identify aneurysms at higher risk of incomplete occlusion, guiding device use, sizing, and treatment. However, further refinement and external validation are needed before clinical use.

In comparing the WEB device to traditional endovascular techniques such as coiling and stenting [10, 12, 20], it is essential to consider both these treatment options’ efficacy and risk profiles. While ATENA, CLARITY, ARETA, and other studies reported higher rates of immediate post-angiographic complete occlusion with traditional techniques, these approaches also involve greater procedural complexity and associated risks [10–12, 20]. The WEB device offers a single-step, intrasaccular solution that rarely requires adjunctive devices (used in only 6% of our cases), though when used, they improved occlusion, especially in unruptured aneurysms. This suggests adjunctive devices are necessary for optimal occlusion in complex cases, making the WEB advantageous for treating challenging aneurysms. However, the WEB device is typically used for aneurysms of different sizes and morphologies than those in the ATENA and ARETA studies, often showing a lower dome-to-neck ratio, which may explain the lower immediate occlusion rates seen.

Furthermore, although the relatively lower occlusion rates with the WEB device may raise concerns, the risk of rebleeding is virtually nonexistent, even when angiographic occlusion is incomplete [9]. This does not mean that incomplete occlusion is an acceptable goal. Instead, it highlights that the WEB’s intrasaccular design offers long-lasting protection even with minor residual filling. However, complete occlusion remains the ideal standard for aneurysm treatment [35].

Finally, to our knowledge, this is the first and largest cohort study to evaluate predictors of immediate post-angiographic occlusion outcomes for the WEB device in treating both ruptured and unruptured intracranial aneurysms. Our results show that aneurysm shape, patient features, and treatment methods greatly affect immediate occlusion rates. While machine learning models have potential in predicting these outcomes, especially in emergency cases like ruptured aneurysms, they need more validation. Achieving optimal results with the WEB device depends on careful patient selection and customized treatment plans, which include precise device sizing and correct placement, as poor sizing can lower complete occlusion rates and require additional devices. Understanding these technical details is key to fully unlocking the WEB’s potential, which, even with less-than-ideal angiographic results, can still provide a reliable and safe treatment option for many patients.

### Limitations and strengths

This study's strengths include a large international sample, comprehensive demographic, clinical, and procedural variables analysis, and robust machine learning models. Multilevel logistic regression was used to account for site differences. However, the retrospective design may introduce bias, with limitations such as incomplete data on aneurysm characteristics, procedural details, and follow-up timing. Although follow-up RROC grades and retreatment data were collected, they were not analyzed in this study, which focused on immediate occlusion outcomes; these data have been discussed in previous manuscripts [24, 36–38]. The study population was primarily from tertiary centers, limiting generalizability. While associations were identified, causal relationships and direct comparisons with other treatment modalities were not established. Despite these limitations, the study provides valuable insights into factors influencing WEB device outcomes, particularly in ruptured aneurysms, and offers a foundation for personalized risk assessment in aneurysm treatment.

## Conclusion

This multicenter study reveals higher immediate complete occlusion rates for the WEB device (38.3% ruptured, 32.8% unruptured aneurysms) than previously reported, though lower than traditional techniques. Larger neck diameter, daughter sacs, emanating branches, smoking history, and higher pre-treatment mRS predict incomplete occlusion, while bifurcation aneurysms and posterior circulation location favor better outcomes. Despite lower occlusion rates, near-zero rebleeding risk suggests immediate complete occlusion may be less critical for WEB devices. Machine learning models show promise for clinical decision support. The WEB device remains viable for complex aneurysms, warranting careful patient selection. Future research should address its long-term outcomes and comparative effectiveness, particularly in ruptured aneurysms.

## Supplementary Information

Below is the link to the electronic supplementary material.

Supplementary file1 (JPG 196 KB)

Supplementary file2 (JPG 197 KB)

Supplementary file3 (JPG 872 KB)

Supplementary file4 (JPG 934 KB)

Supplementary file5 (JPG 219 KB)

Supplementary file6 (JPG 240 KB)

Supplementary file7 (JPG 737 KB)

Supplementary file8 (JPG 2468 KB)

Supplementary file9 (JPG 275 KB)

Supplementary file10 (JPG 440 KB)

Supplementary file11 (JPG 273 KB)

Supplementary file12 (JPG 278 KB)

Supplementary file13 (DOCX 20.2 KB)

## Data Availability

Data generated or analyzed during the study are available from the corresponding author by request.

## Declarations

### Clinical trial

Clinical trial number: not applicable.

### Previous presentations

This study was presented as an e-poster at the AANS in Chicago on May 4th, 2024.

## References

[1] Ding YH, Lewis DA, Kadirvel R et al (2011) The woven endobridge: a new aneurysm occlusion device. AJNR Am J Neuroradiol 32:607–611 21330397 10.3174/ajnr.A2399 PMC8013102

[2] Cagnazzo F, Cloft HJ, Lanzino G et al (2023) WEB (Woven endobridge) device for intracranial aneurysm treatment: technical, radiological, and clinical findings in a consecutive North American cohort. Acta Neurochir (Wien) 165:2077–2086 37365349 10.1007/s00701-023-05668-6

[3] Asnafi S, Rouchaud A, Pierot L et al (2016) Efficacy and safety of the woven endobridge (WEB) device for the treatment of intracranial aneurysms: a systematic review and meta-analysis. AJNR Am J Neuroradiol 37:2287–2292 27516237 10.3174/ajnr.A4900 PMC7963876

[4] Arthur AS, Molyneux A, Coon AL et al (2019) The safety and effectiveness of the woven endobridge (WEB) system for the treatment of wide-necked bifurcation aneurysms: final 12-month results of the pivotal WEB intrasaccular therapy (WEB-IT) study. J Neurointerv Surg 11:924–930 30992395 10.1136/neurintsurg-2019-014815 PMC6824604

[5] Fiorella D, Molyneux A, Coon A et al (2023) Safety and effectiveness of the woven endobridge (WEB) system for the treatment of wide necked bifurcation aneurysms: final 5 year results of the pivotal WEB Intra-saccular Therapy study (WEB-IT). J Neurointerv Surg 15:1175–1180 37355252 10.1136/jnis-2023-020611 PMC10715507

[6] Hassankhani A, Ghozy S, Amoukhteh M et al (2023) Long-term outcomes of the Woven EndoBridge device for treatment of intracranial aneurysms: A systematic review and meta-analysis. Interv Neuroradiol 15910199231184524. 10.1177/15910199231184524 10.1177/15910199231184524 PMC13294524 37357734

[7] Kewlani B, Ryan DJ, Henry J et al (2023) A single centre retrospective analysis of short- and medium-term outcomes using the woven endobridge (WEB) device and identification of the device-to-aneurysm volume ratio as a potential predictor of aneurysm occlusion status. Interv Neuroradiol 29:393–401 35404152 10.1177/15910199221092578 PMC10399511

[8] Raj R, Rautio R, Pekkola J et al (2019) Treatment of ruptured intracranial aneurysms using the woven endobridge device: a two-center experience. World Neurosurg 123:e709–e716 30576812 10.1016/j.wneu.2018.12.010

[9] Essibayi MA, Lanzino G, Brinjikji W (2021) Safety and efficacy of the woven endobridge device for treatment of ruptured intracranial aneurysms: a systematic review and meta-analysis. AJNR Am J Neuroradiol 42:1627–1632 34117016 10.3174/ajnr.A7174 PMC8423055

[10] Pierot L, Cognard C, Ricolfi F et al (2010) Immediate anatomic results after the endovascular treatment of ruptured intracranial aneurysms: analysis in the CLARITY series. AJNR Am J Neuroradiol 31:907–911 20075090 10.3174/ajnr.A1954 PMC7964183

[11] Pineda-Castillo SA, Jones ER, Laurence KA et al (2024) Systematic review and meta-analysis of endovascular therapy effectiveness for unruptured saccular intracranial aneurysms. Stroke Vasc Interv Neurol 4:e001118 38846323 10.1161/SVIN.123.001118 PMC11152505

[12] Pierot L, Spelle L, Vitry F et al (2010) Immediate anatomic results after the endovascular treatment of unruptured intracranial aneurysms: analysis of the ATENA series. AJNR Am J Neuroradiol 31:140–144 19729540 10.3174/ajnr.A1745 PMC7964053

[13] Celik E, Ozpeynirci Y, Liebig T et al (2022) Comparison of angiographic outcomes and complication rates of WEB embolization and coiling for treatment of unruptured basilar tip aneurysms. Sci Rep 12:10899 35764798 10.1038/s41598-022-15113-w PMC9240056

[14] Mortezaei A, Yazdanian F, Mirahmadi Eraghi M et al (2025) Retreatment rate and strategies for recurrent and residual aneurysms after woven endobridge (WEB) treatment: a comprehensive systematic review and meta-analysis. Neurosurg Rev 48:400 40316859 10.1007/s10143-025-03532-y PMC12048415

[15] Mascitelli JR (2024) Management of wide-neck aneurysms in 2024: how does one make the best treatment decision when there are so many good options? J Neurointerv Surg 16:433–434 38653525 10.1136/jnis-2024-021732

[16] Nania A, Gatt S, Banerjee R et al (2023) WEB vs coiling in ruptured aneurysms: a propensity score matched comparison of safety and efficacy. Interv Neuroradiol 29:402–407 35379037 10.1177/15910199221092241 PMC10399506

[17] Kashkoush A, El-Abtah ME, Srivatsa S et al (2023) Comparative effectiveness of stent-assisted coiling and woven endobridge embolization for the treatment of unruptured wide-neck bifurcation intracranial aneurysms. J Neurosurg 138:1487–1493 36334292 10.3171/2022.10.JNS221138

[18] Kabbasch C, Goertz L, Siebert E et al (2019) Comparison of WEB embolization and coiling in unruptured intracranial aneurysms: safety and efficacy based on a propensity score analysis. World Neurosurg 126:e937–e943 30862582 10.1016/j.wneu.2019.03.016

[19] Pierot L, Szikora I, Barreau X et al (2023) Aneurysm treatment with the woven endobridge (WEB) device in the combined population of two prospective, multicenter series: 5-year follow-up. J Neurointerv Surg 15:552–557 35803731 10.1136/neurintsurg-2021-018414 PMC10314010

[20] Pierot L, Barbe C, Herbreteau D et al (2021) Immediate post-operative aneurysm occlusion after endovascular treatment of intracranial aneurysms with coiling or balloon-assisted coiling in a prospective multicenter cohort of 1189 patients: analysis of recanalization after endovascular treatment of intracranial aneurysm (ARETA) study. J Neurointerv Surg 13:918–923 33443137 10.1136/neurintsurg-2020-017012

[21] Lubicz B, Klisch J, Gauvrit JY et al (2014) Web-dl endovascular treatment of wide-neck bifurcation aneurysms: short- and midterm results in a European study. AJNR Am J Neuroradiol 35:432–438 24457823 10.3174/ajnr.A3869 PMC7964738

[22] Liang F, Zhang Y, Guo F et al (2018) Use of pipeline embolization device for posterior circulation aneurysms: single-center experiences with comparison with anterior circulation aneurysms. World Neurosurg 112:e683–e690 29410337 10.1016/j.wneu.2018.01.129

[23] Zhang X-J, Hao W-L, Zhang D-H et al (2019) Asymmetrical middle cerebral artery bifurcations are more vulnerable to aneurysm formation. Sci Rep 9:15255 31649321 10.1038/s41598-019-51734-4 PMC6813347

[24] Adeeb N, Dibas M, Diestro JDB et al (2022) Multicenter study for the treatment of sidewall versus bifurcation intracranial aneurysms with use of Woven EndoBridge (WEB). Radiology 304:372–382 35438564 10.1148/radiol.212006

[25] Cortese J, Caroff J, Chalumeau V et al (2022) Determinants of cerebral aneurysm occlusion after embolization with the WEB device: a single-institution series of 215 cases with angiographic follow-up. J Neurointerv Surg 15:446–451 35428742 10.1136/neurintsurg-2022-018780

[26] Adeeb N, Griessenauer CJ, Dmytriw AA et al (2018) Risk of branch occlusion and ischemic complications with the Pipeline Embolization Device in the treatment of posterior circulation aneurysms. AJNR Am J Neuroradiol 39:1303–1309 29880475 10.3174/ajnr.A5696 PMC7655425

[27] Gonzalez NR, Dusick JR, Duckwiler G et al (2010) Endovascular coiling of intracranial aneurysms in elderly patients: report of 205 treated aneurysms. Neurosurgery 66:714 20190665 10.1227/01.NEU.0000367451.59090.D7

[28] Maragkos GA, Ascanio LC, Salem MM et al (2019) Predictive factors of incomplete aneurysm occlusion after endovascular treatment with the Pipeline embolization device. Epub ahead of print 26 April 2019. 10.3171/2019.1.JNS183226 10.3171/2019.1.JNS183226 31026827

[29] Ferns SP, Majoie CBLM, Sluzewski M et al (2010) Late adverse events in coiled ruptured aneurysms with incomplete occlusion at 6-month angiographic follow-up. AJNR Am J Neuroradiol 31:464–469 19833795 10.3174/ajnr.A1841 PMC7963999

[30] Savarraj JPJ, Parsha K, Hergenroeder GW et al (2017) Systematic model of peripheral inflammation after subarachnoid hemorrhage. Neurology 88:1535–1545 28314864 10.1212/WNL.0000000000003842 PMC5395070

[31] de Oliveira Manoel AL, Macdonald RL (2018) Neuroinflammation as a target for intervention in subarachnoid hemorrhage. Front Neurol. 10.3389/fneur.2018.00292 29770118 10.3389/fneur.2018.00292 PMC5941982

[32] Futchko J, Starr J, Lau D et al (2018) Influence of smoking on aneurysm recurrence after endovascular treatment of cerebrovascular aneurysms. J Neurosurg. 10.3171/2016.12.JNS161625 28644100 10.3171/2016.12.JNS161625

[33] Goyal N, Hoit D, DiNitto J et al (2020) How to WEB: a practical review of methodology for the use of the woven endobridge. J Neurointerv Surg 12:512–520 32005760 10.1136/neurintsurg-2019-015506 PMC7231463

[34] Mastorakos P, Naamani KE, Adeeb N et al (2024) Predictors of aneurysm obliteration in patients treated with the WEB device: results of a multicenter retrospective study. AJNR Am J Neuroradiol 45:906–911 38977286 10.3174/ajnr.A8324 PMC11286027

[35] Hoh BL, Ko NU, Amin-Hanjani S et al (2023) Guideline for the management of patients with aneurysmal subarachnoid hemorrhage: a guideline from the American heart association/American stroke association. Stroke 54. Epub ahead of print July 2023. 10.1161/STR.0000000000000436 10.1161/STR.0000000000000436 37212182

[36] Essibayi MA, Jabal MS, Jamil H et al (2025) Prediction of persistent incomplete occlusion of intracranial aneurysms treated with woven EndoBridge device. Neurosurg Rev 48:314 40119209 10.1007/s10143-025-03439-8 PMC11928387

[37] Dmytriw AA, Diestro JDB, Dibas M et al (2022) International study of intracranial aneurysm treatment using Woven EndoBridge: results of the WorldWideWEB consortium. Stroke 53:e47–e49 34915737 10.1161/STROKEAHA.121.037609 PMC8792251

[38] Saliou G, Salim HA, Musmar B et al (2025) Higher risk of recurrence in partially thrombosed cerebral aneurysms post-WEB (Woven EndoBridge) device treatment: insights from the WorldWideWEB Consortium registry. J Neurointerv Surg jnis-2024–022628. 10.1136/jnis-2024-022628 10.1136/jnis-2024-022628 PMC13217109 40306928

