# Supplementary figures and images for: Factors influencing immediate post-angiographic occlusion outcomes in intracranial aneurysms treated with the woven endobridge device: a multi-center analysis and predictive model from the WorldWideWEB consortium
Source: Neurosurg Rev. 2025 Dec 2;49(1):36. doi: 10.1007/s10143-025-03928-w (PMC12669363; doi:10.1007/s10143-025-03928-w)

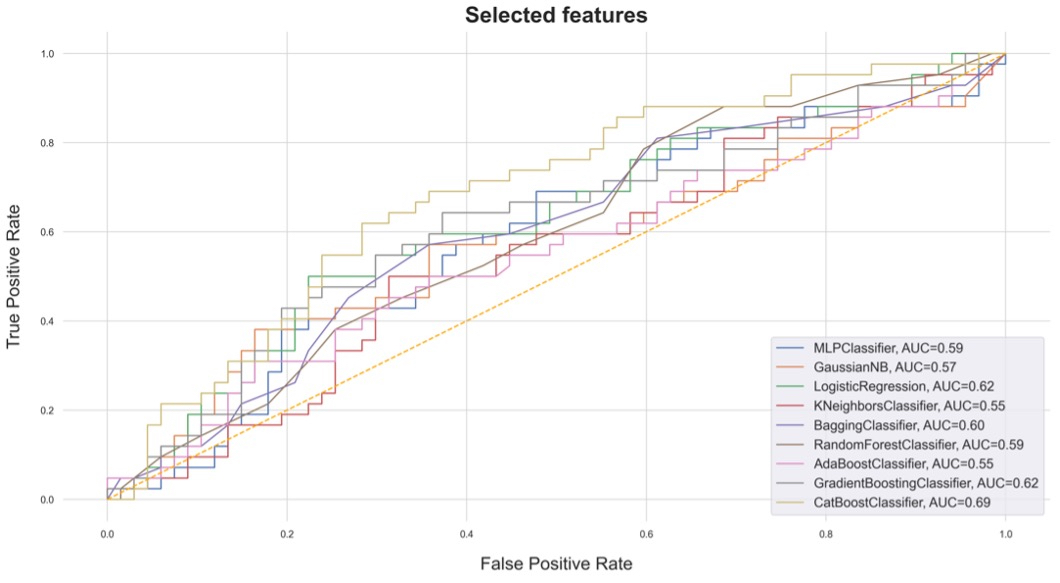

Supplement: Supplementary file 1 — Supplementary file1 (JPG 196 KB) [file 10143_2025_3928_MOESM1_ESM.jpg]

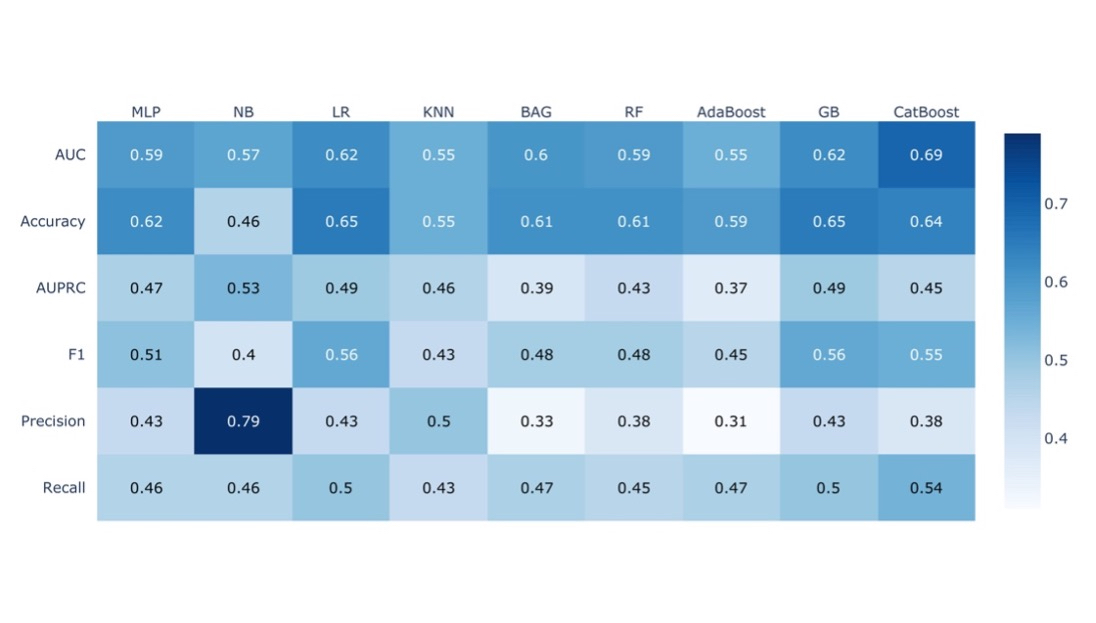

Supplement: Supplementary file 2 — Supplementary file2 (JPG 197 KB) [file 10143_2025_3928_MOESM2_ESM.jpg]

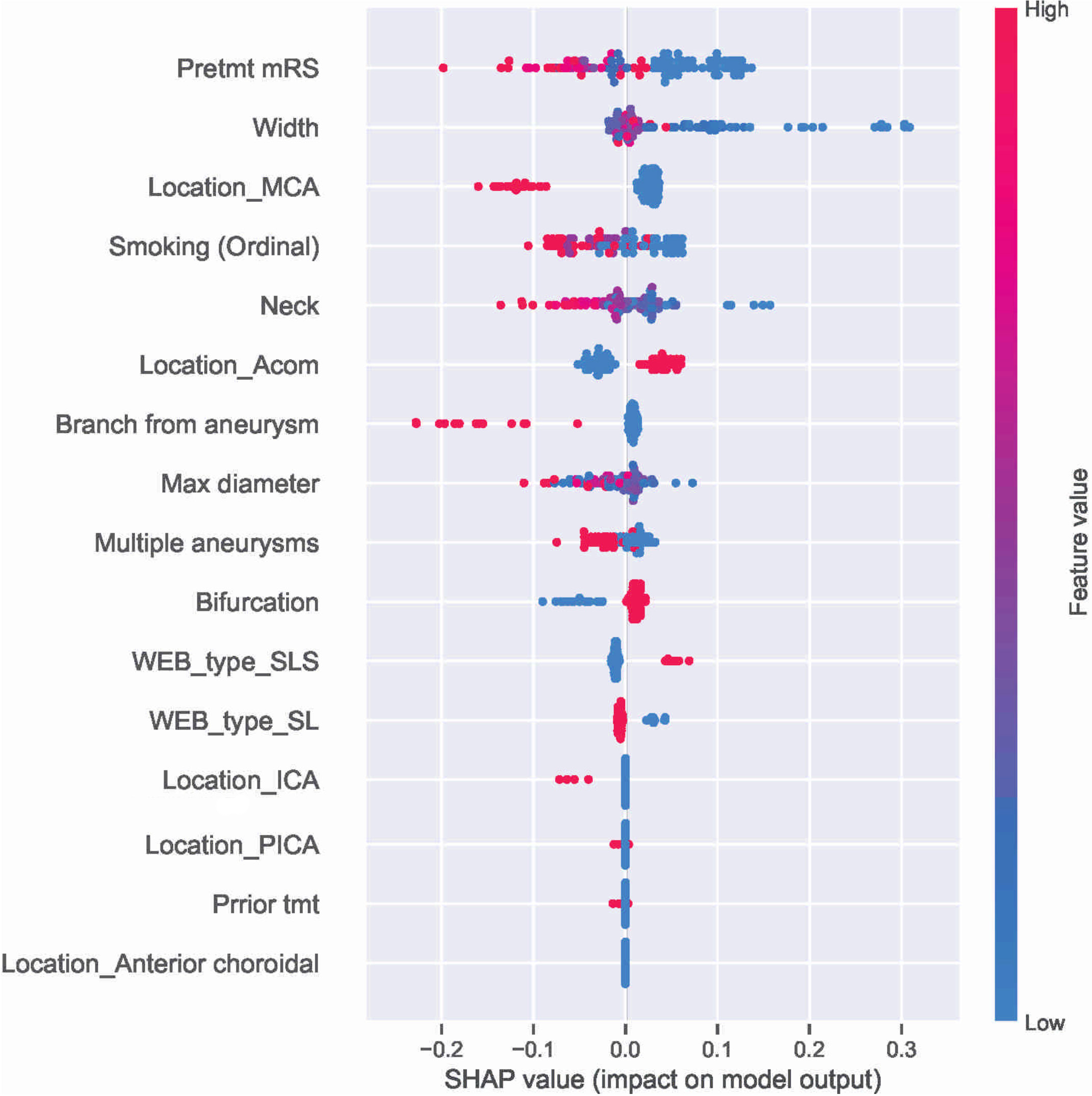

Supplement: Supplementary file 3 — Supplementary file3 (JPG 872 KB) [file 10143_2025_3928_MOESM3_ESM.jpg]

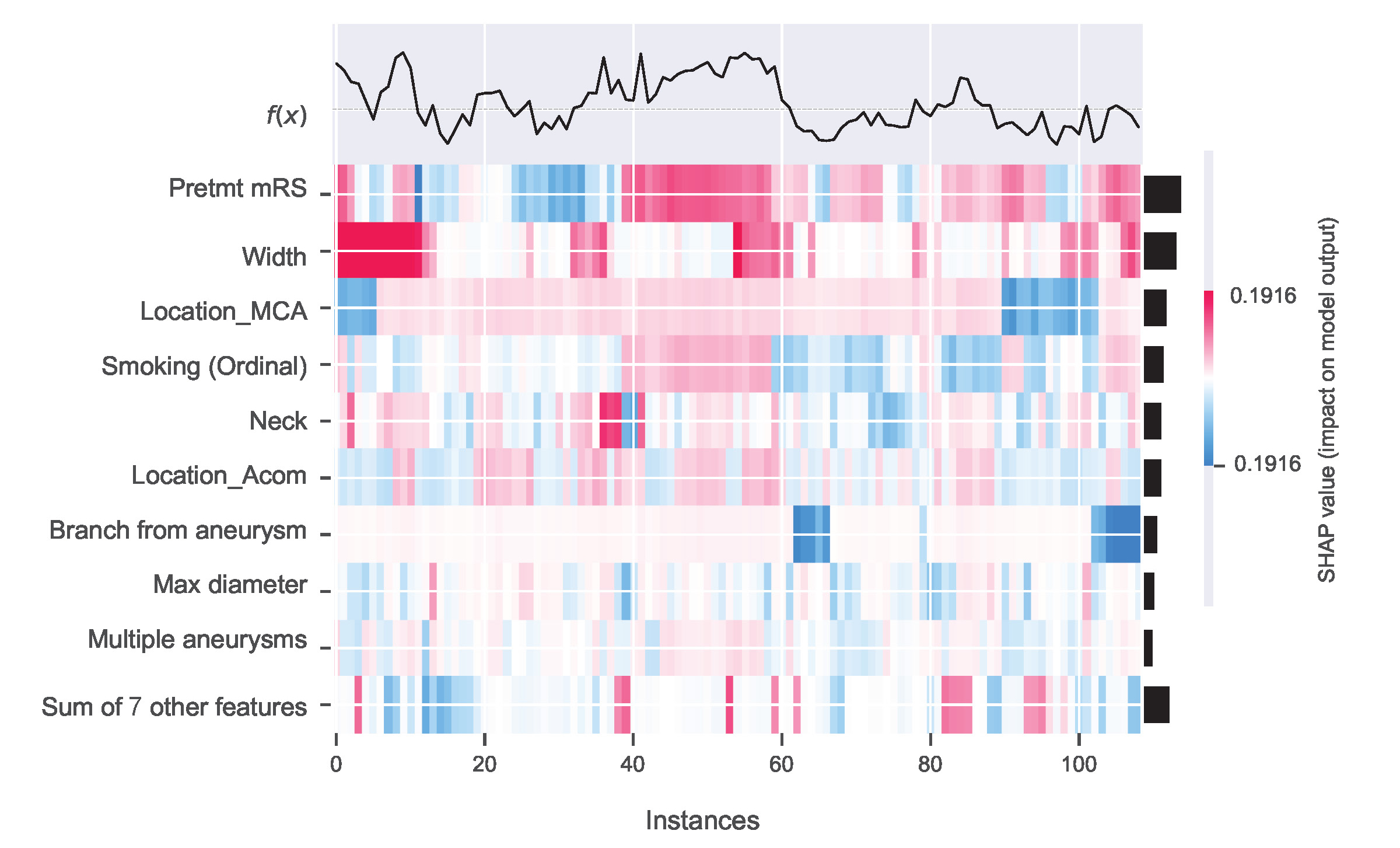

Supplement: Supplementary file 4 — Supplementary file4 (JPG 934 KB) [file 10143_2025_3928_MOESM4_ESM.jpg]

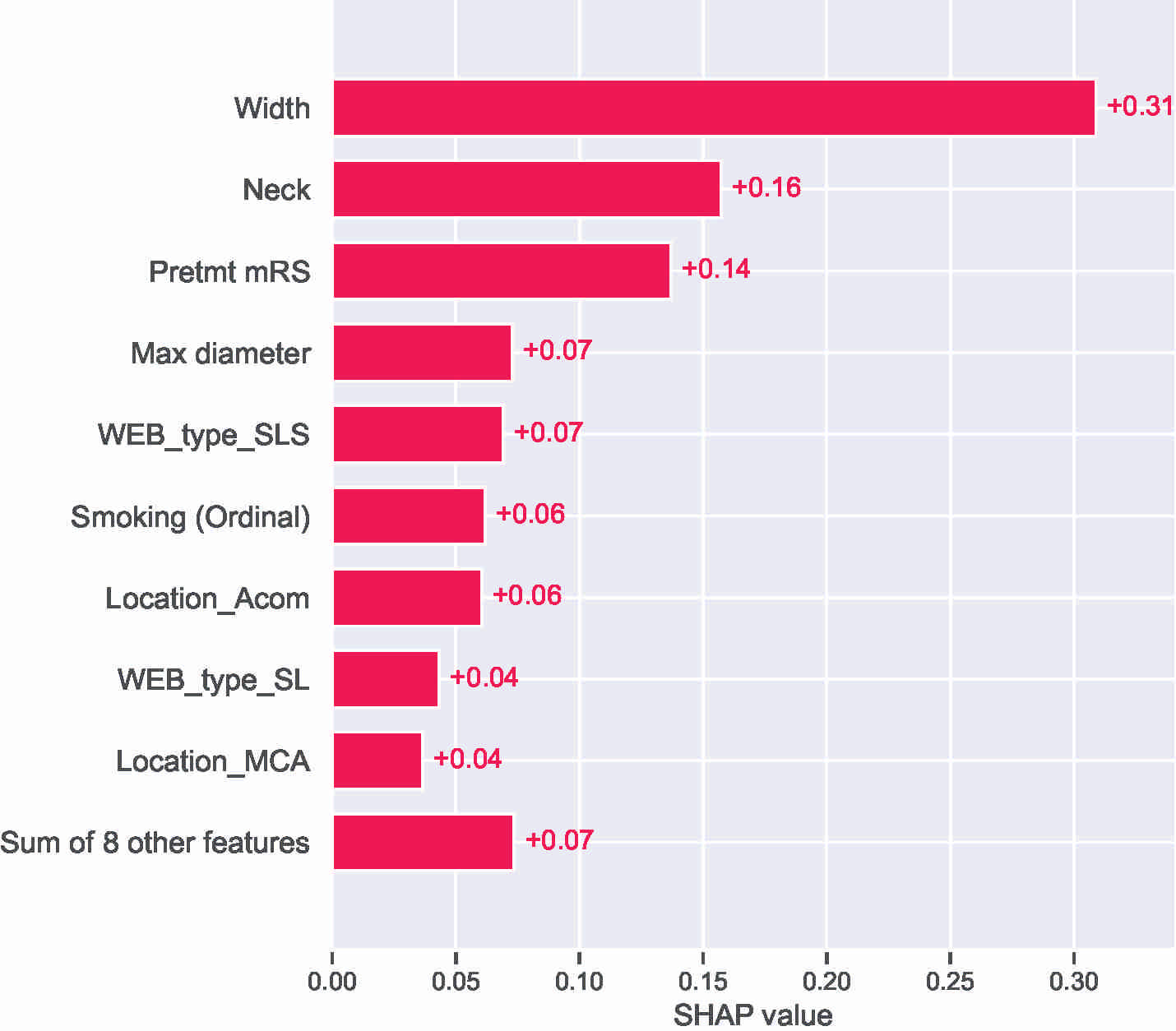

Supplement: Supplementary file 5 — Supplementary file5 (JPG 219 KB) [file 10143_2025_3928_MOESM5_ESM.jpg]

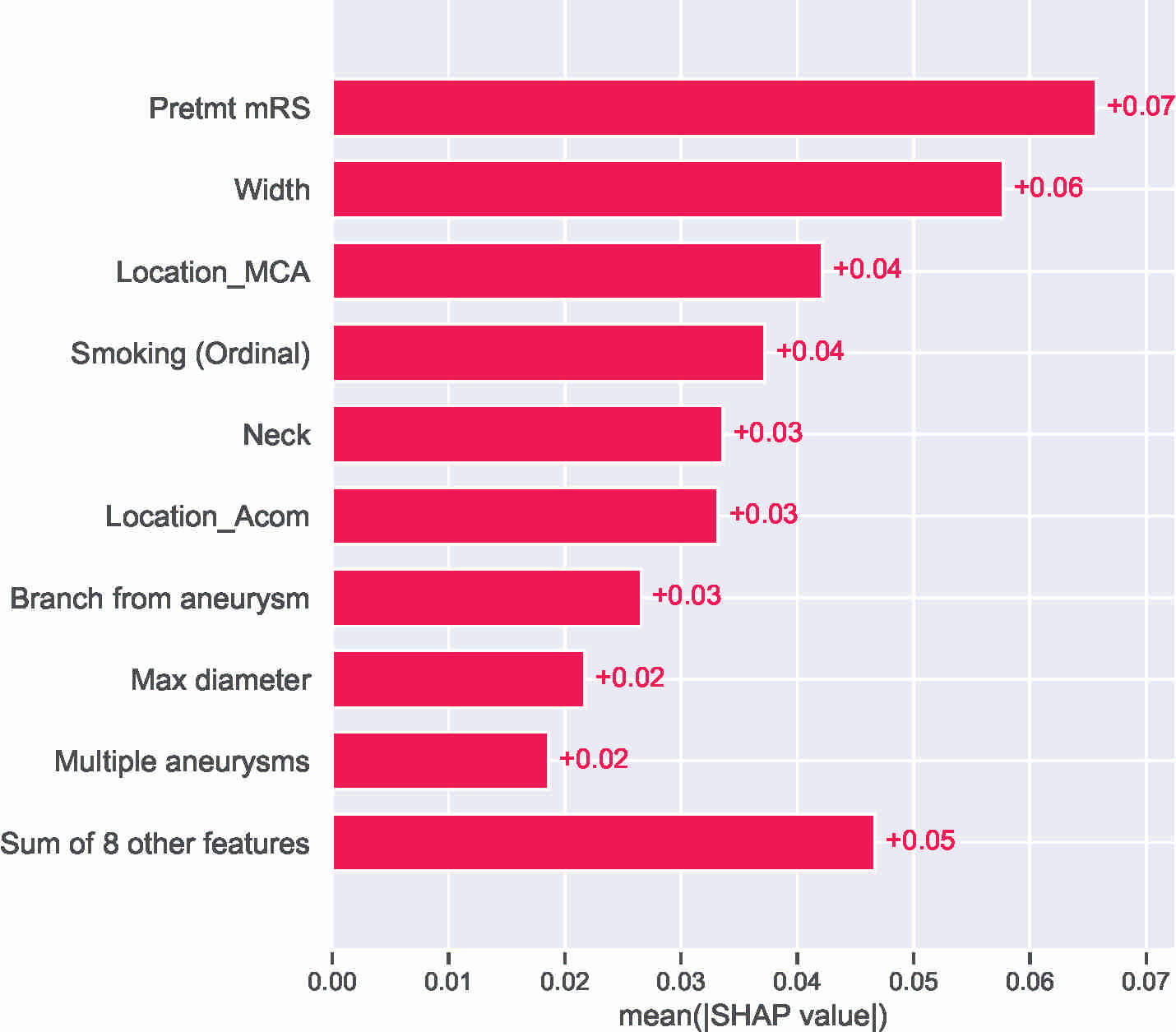

Supplement: Supplementary file 6 — Supplementary file6 (JPG 240 KB) [file 10143_2025_3928_MOESM6_ESM.jpg]

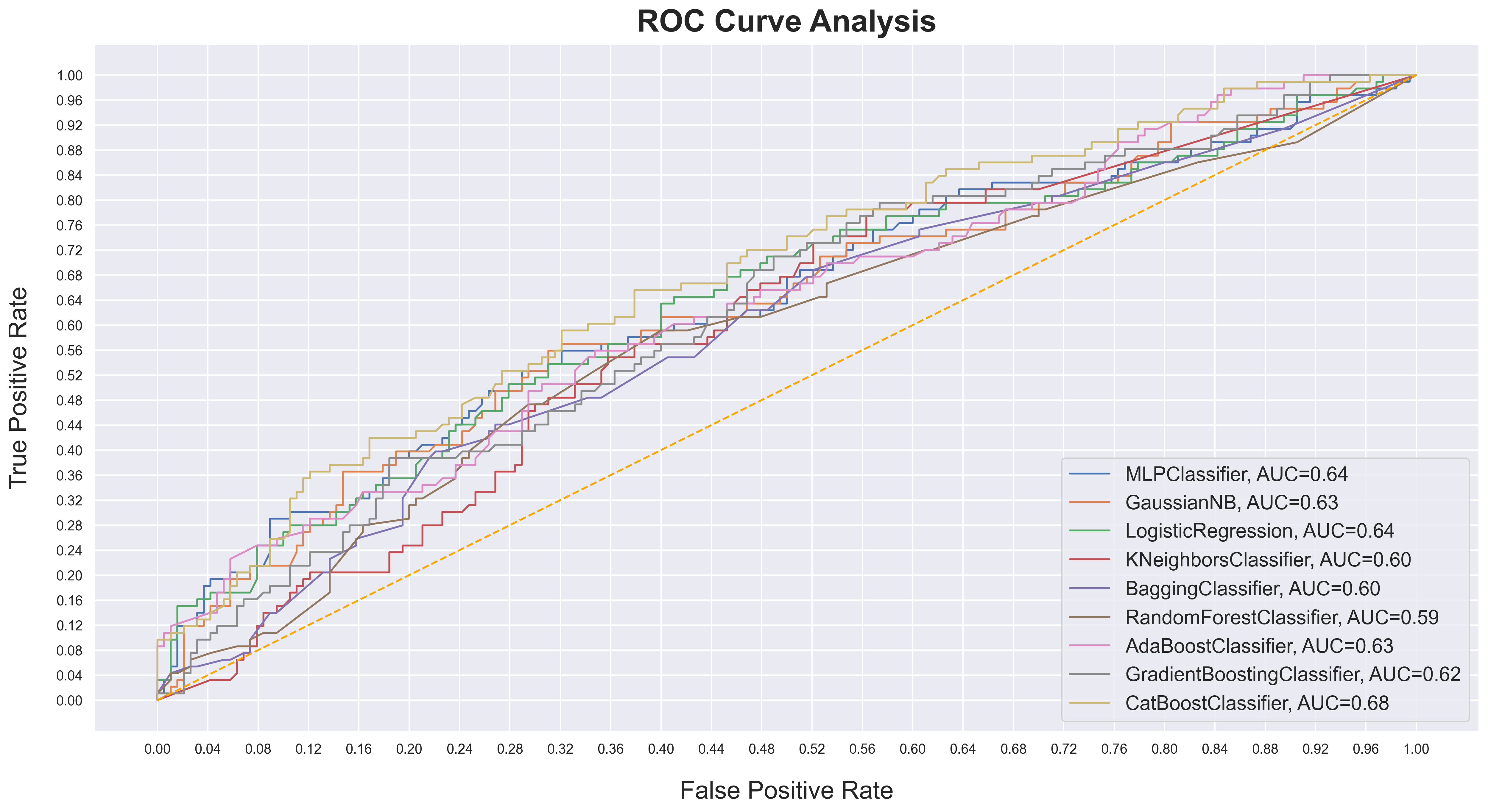

Supplement: Supplementary file 7 — Supplementary file7 (JPG 737 KB) [file 10143_2025_3928_MOESM7_ESM.jpg]

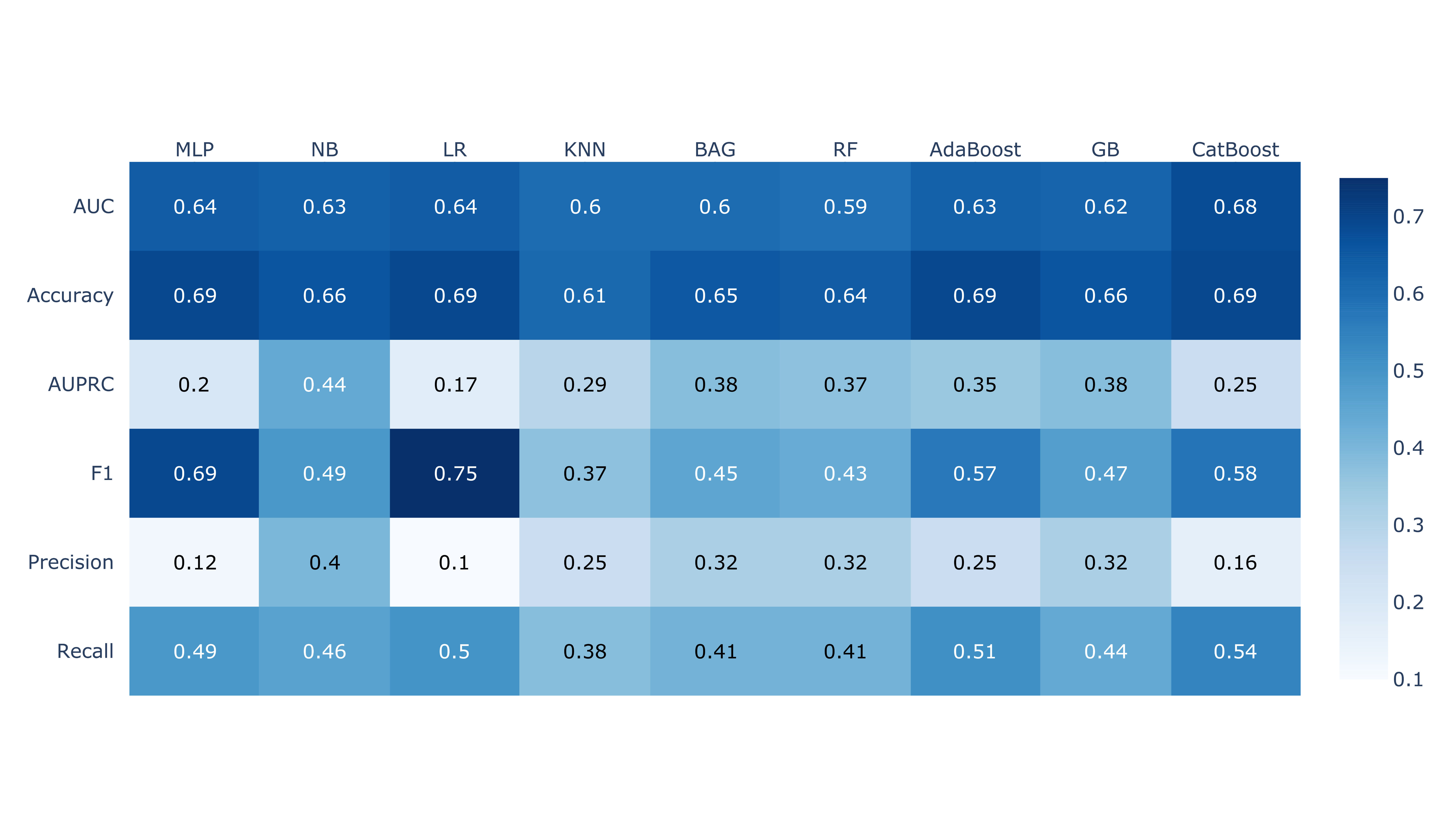

Supplement: Supplementary file 8 — Supplementary file8 (JPG 2468 KB) [file 10143_2025_3928_MOESM8_ESM.jpg]

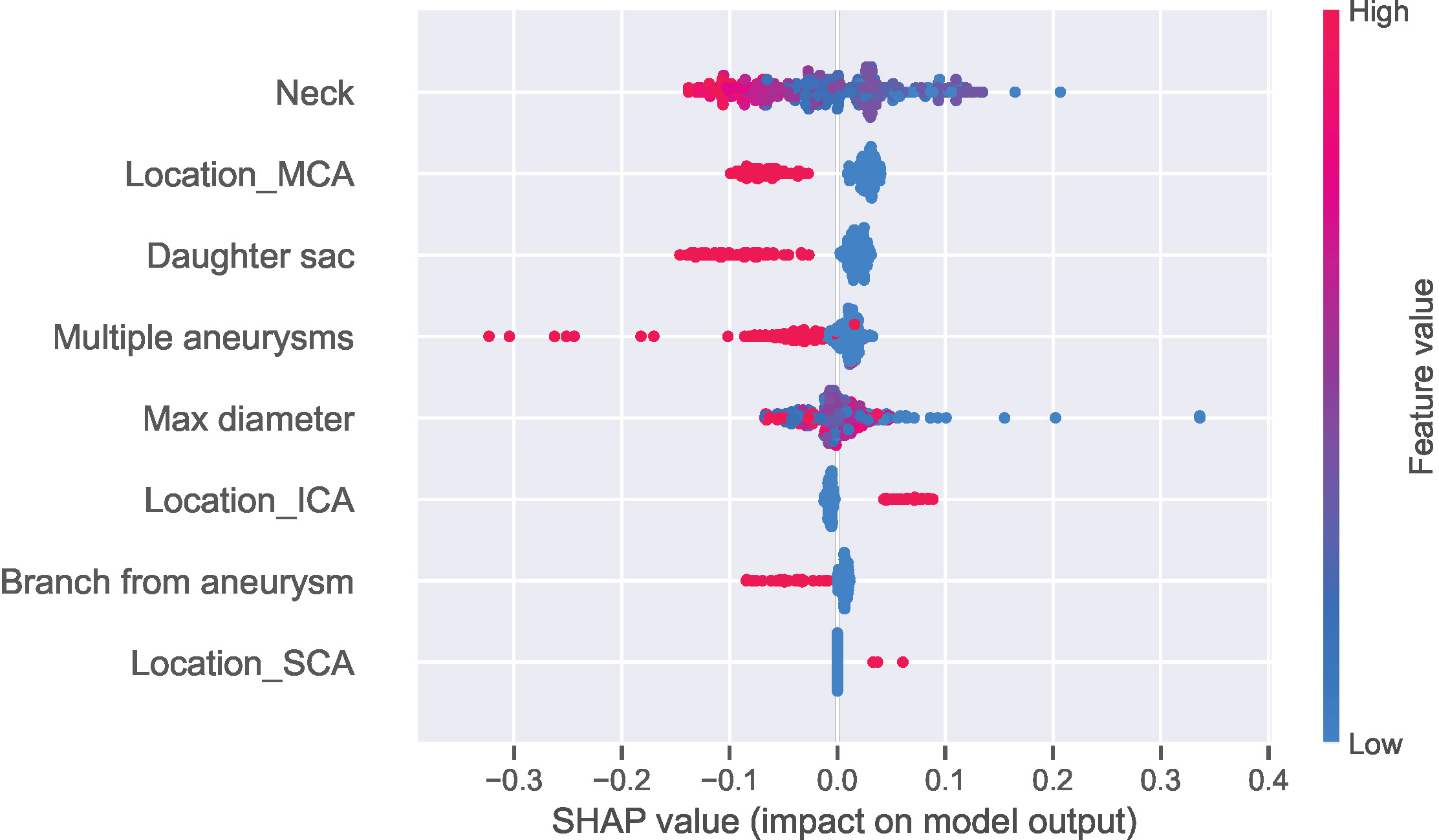

Supplement: Supplementary file 9 — Supplementary file9 (JPG 275 KB) [file 10143_2025_3928_MOESM9_ESM.jpg]

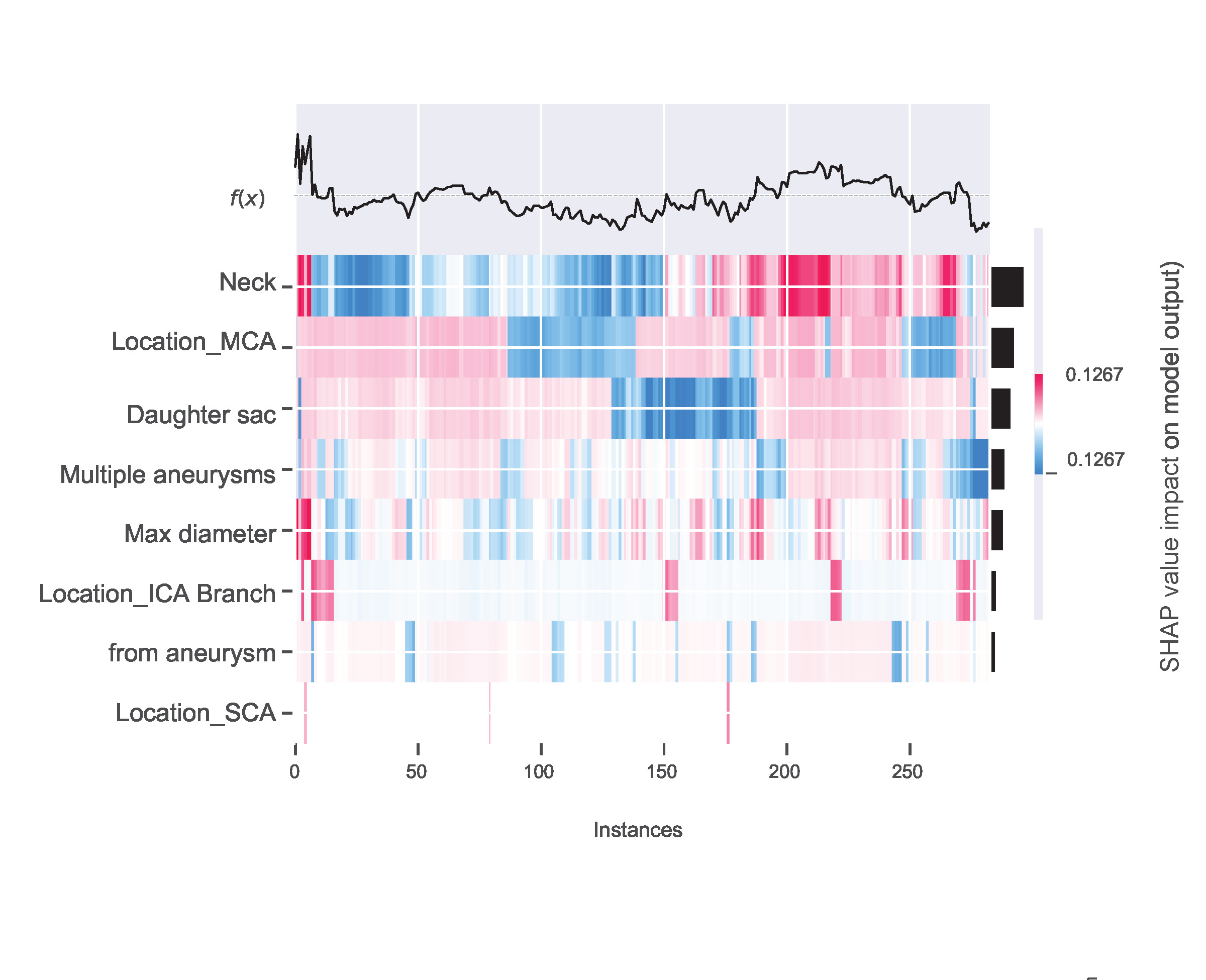

Supplement: Supplementary file 10 — Supplementary file10 (JPG 440 KB) [file 10143_2025_3928_MOESM10_ESM.jpg]

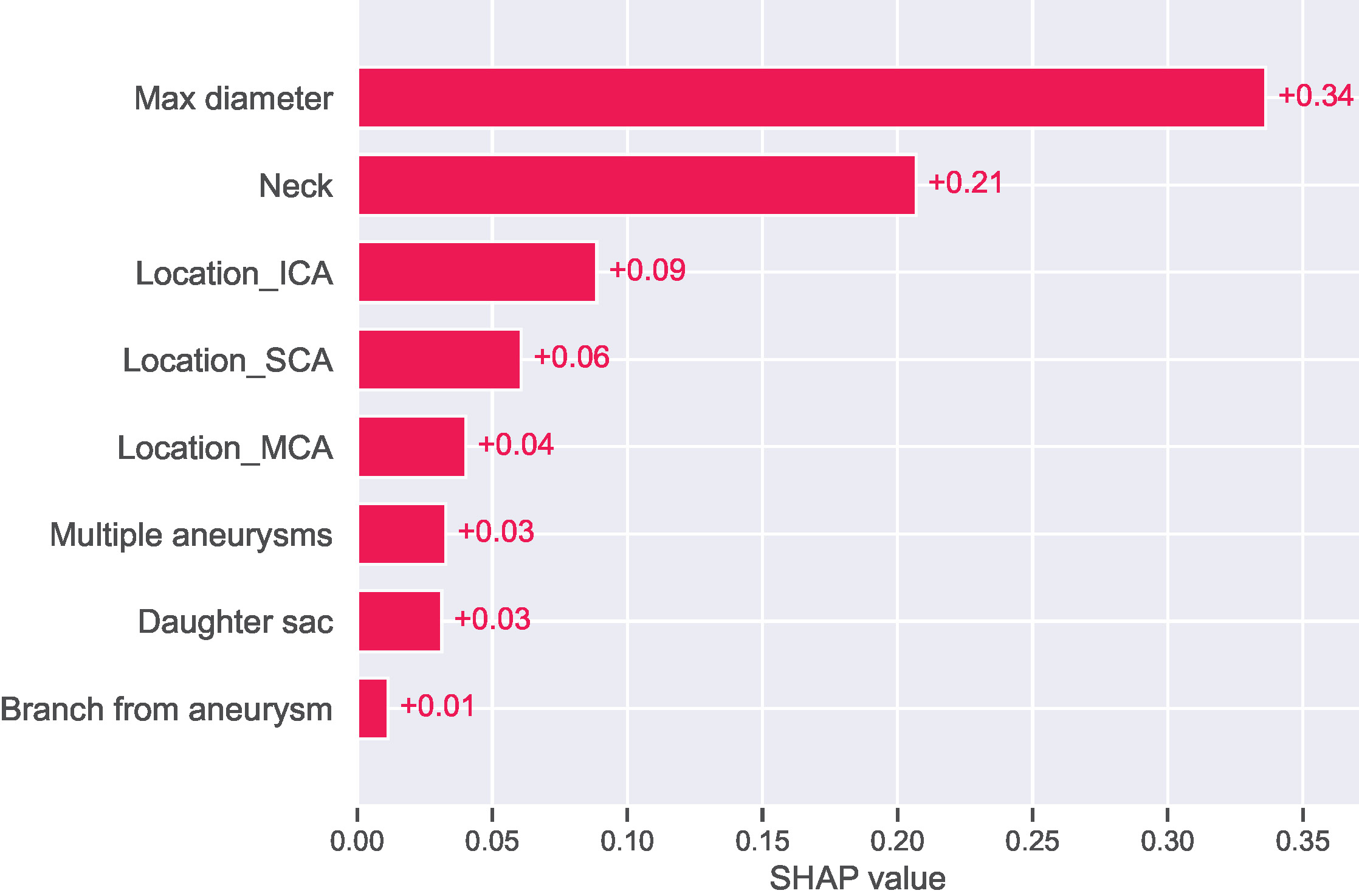

Supplement: Supplementary file 11 — Supplementary file11 (JPG 273 KB) [file 10143_2025_3928_MOESM11_ESM.jpg]

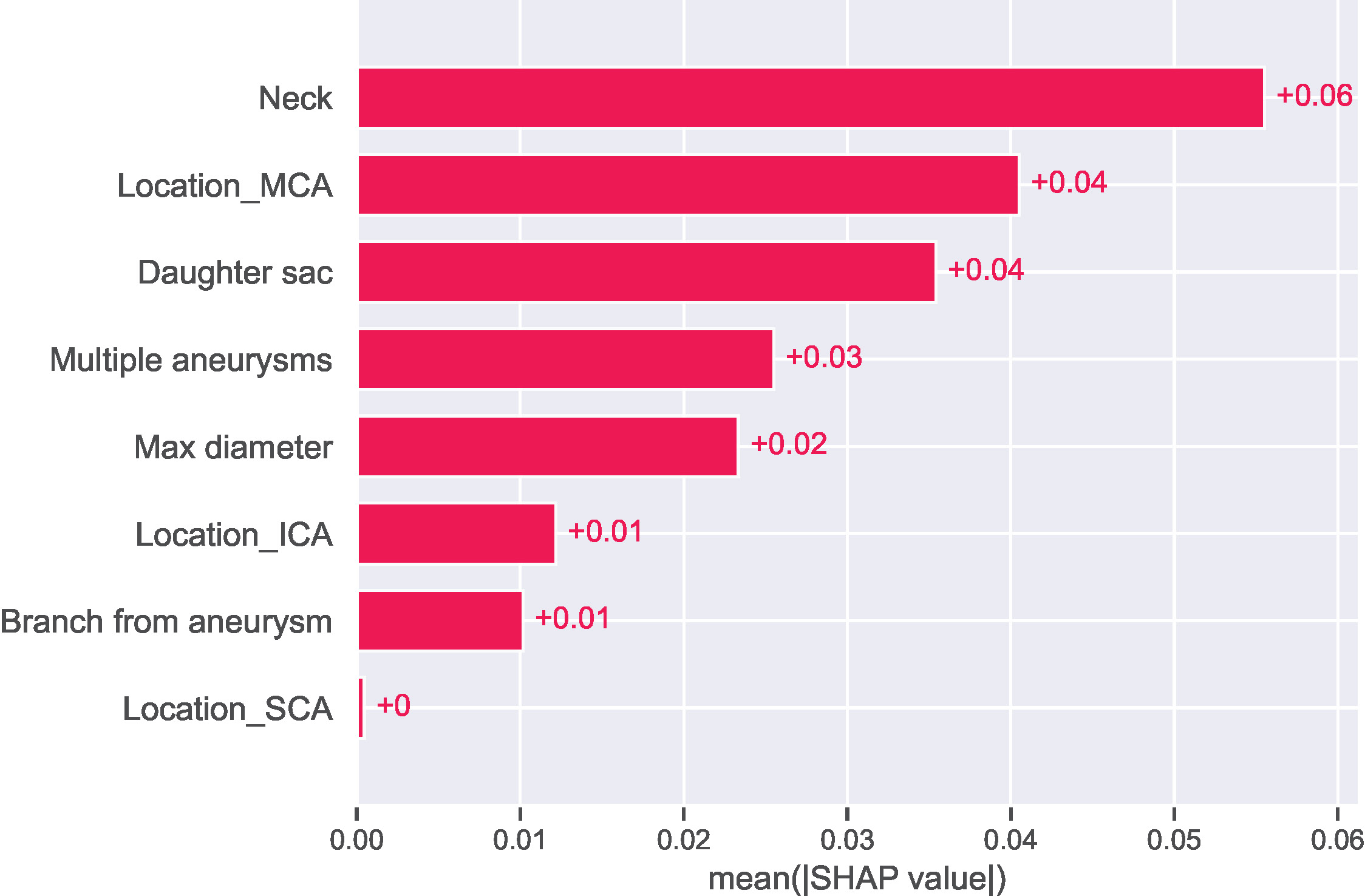

Supplement: Supplementary file 12 — Supplementary file12 (JPG 278 KB) [file 10143_2025_3928_MOESM12_ESM.jpg]
