# Supplementary material for: Factors influencing immediate post-angiographic occlusion outcomes in intracranial aneurysms treated with the woven endobridge device: a multi-center analysis and predictive model from the WorldWideWEB consortium
Source: Neurosurg Rev. 2025 Dec 2;49(1):36. doi: 10.1007/s10143-025-03928-w (PMC12669363; doi:10.1007/s10143-025-03928-w)
Supplement: Supplementary file 13 — Supplementary file13 (DOCX 20.2 KB) [file 10143_2025_3928_MOESM13_ESM.docx]

**SUPPLEMENTARY**

**Supplemental Table 1.** Multilevel Ordinal Regression to predict worse immediate post-angiographic occlusion outcomes after WEB deployment stratified by Rupture status

|  | Ruptured Aneurysms (n= 436) | | Unruptured Aneurysms (n= 1129) | |
| --- | --- | --- | --- | --- |
|  | Odds ratio (95% CI)^1^ | p-value | Odds ratio (95% CI)^1^ | p-value |
| Demographics | | | | |
| Pre-Treatment mRS Score | 1.04 [0.89, 1.21] | 0.64 | 1.01 [0.81, 1.26] | 0.92 |
| Age | 1.01 [0.99, 1.02] | 0.50 | 1.01 [0.99, 1.02] | 0.35 |
| Gender (Male) | 0.64 [0.40, 1.01] | 0.06 | 0.85 [0.61, 1.17] | 0.31 |
| History of Smoking | 1.95 [1.19, 3.19] | **0.008** | 0.88 [0.64, 1.22] | 0.45 |
| Aneurysm Characteristics | | | | |
| Bifurcation Aneurysm | 0.95 [0.49, 1.85] | 0.89 | 1.11 [0.96, 1.28] | 0.68 |
| Neck Diameter | 1.35 [1.08, 1.70] | **0.008** | 1.05 [0.93, 1.18] | 0.14 |
| Maximal Diameter | 1.17 [0.93, 1.49] | 0.18 | 1.26 [0.80, 1.99] | 0.47 |
| Branch from Aneurysm | 1.62 [0.79, 3.30] | 0.19 | 1.06 [0.72, 1.55] | 0.32 |
| Posterior Circulation | 1.27 [0.66, 2.45] | 0.47 | 0.96 [0.91, 1.01] | 0.77 |
| Aneurysm Height | 0.93 [0.77, 1.10] | 0.39 | 0.99 [0.87, 1.12] | 0.14 |
| Aneurysm Width | 0.94 [0.76, 1.15] | 0.55 | 1.23 [0.84, 1.79] | 0.86 |
| Daughter Sac | 0.87 [0.53, 1.42] | 0.59 | 1.11 [0.96, 1.28] | 0.28 |
| Treatment Characteristics | | | | |
| Prior Treatment | 1.45 [0.59, 3.56] | 0.41 | 1.02 [0.56, 1.86] | 0.94 |
| Use of Adjunct Device | 0.71 [0.28, 1.84] | 0.48 | 0.89 [0.50, 1.55] | 0.67 |
| WEB Device Type |  |  |  |  |
| WEB Device Type (DL) | [Reference] |  | [Reference] |  |
| WEB Device Type (SL) | 2.86 [0.13, 62.99] | 0.51 | 1.52 [0.29, 8.00] | 0.62 |
| WEB Device Type (SLS) | 1.47 [0.06, 34.15] | 0.81 | 1.27 [0.23, 6.99] | 0.78 |

The dependent variable “Immediate post-angiographic occlusion status” has the following order 1 “complete occlusion” | 2 “neck remnant” | 3 “aneurysm remnant”

**Abbreviations: CI**, Confidence Interval; **DL**, Dual-Layer; **mRS**, modified Rankin Scale; **SL**, Single-Layer; **SLS**, Single-Layer Spherical.
